# Systematic evaluation of connectivity map for disease indications

**DOI:** 10.1186/s13073-014-0095-1

**Published:** 2014-12-02

**Authors:** Jie Cheng, Lun Yang, Vinod Kumar, Pankaj Agarwal

**Affiliations:** Value Evidence Analytics, GlaxoSmithKline R&D, UP4335, 1250 S Collegeville Rd, Collegeville, PA 19426 USA; Computational Biology, GlaxoSmithKline R&D, UW2230, 709 Swedeland Road, King of Prussia, PA 19406 USA

## Abstract

**Background:**

Connectivity map data and associated methodologies have become a valuable tool in understanding drug mechanism of action (MOA) and discovering new indications for drugs. One of the key ideas of connectivity map (CMAP) is to measure the connectivity between disease gene expression signatures and compound-induced gene expression profiles. Despite multiple impressive anecdotal validations, only a few systematic evaluations have assessed the accuracy of this aspect of CMAP, and most of these utilize drug-to-drug matching to transfer indications across the two drugs.

**Methods:**

To assess CMAP methodologies in a more direct setting, namely the power of classifying known drug-disease relationships, we evaluated three CMAP-based methods on their prediction performance against a curated dataset of 890 true drug-indication pairs. The disease signatures were generated using Gene Logic BioExpress™ system and the compound profiles were derived from the Connectivity Map database (CMAP, build 02, http://www.broadinstitute.org/CMAP/).

**Results:**

The similarity scoring algorithm called eXtreme Sum (XSum) performs better than the standard Kolmogorov-Smirnov (KS) statistic in terms of the area under curve and can achieve a four-fold enrichment at 0.01 false positive rate level, with AUC = 2.2E-4, *P* value = 0.0035.

**Conclusion:**

Connectivity map can significantly enrich true positive drug-indication pairs given an effective matching algorithm.

**Electronic supplementary material:**

The online version of this article (doi:10.1186/s13073-014-0095-1) contains supplementary material, which is available to authorized users.

## Background

Identifying the correct indications for a drug is important as it is often a surrogate for deciding which patients to treat with the drug, and several computational methods have been described in the literature to aid in this process [1]. Transcriptomic data have been used to build disease-disease [2] or disease-drug relationships that could lead to new drug repositioning hypotheses [3,4]. The problem for any practitioner, however, is to assess the precision of these methods. The desired method should provide relatively high confidence that the first few indications that are predicted for a drug contain at least one that will be validated in clinical trials and make a positive impact on patients.

One of the most important techniques in the space of drug repositioning is connectivity map (CMAP) [5]. CMAP is a database of genome wide transcriptional expression profiles of bioactive small molecules from cultured human cell lines and pattern-matching algorithms that taken together enables the discovery of drug-disease relationships as well as drug mechanism of action [5,6]. Several hypotheses that match therapeutic compounds to new disease indications have been experimentally validated using this approach [7-12].

However, despite numerous impressive anecdotal validations, very few systematic evaluations have been done to estimate the prediction performance of drug-disease relationship. This is because a lot of effort is needed to compile true drug-disease associations and generate large numbers of disease gene signatures across these different disease areas. Most of the earlier research was largely focused on evaluating CMAP methodologies to predict drug-drug relationships [13]. This approach was taken with the implicit hope that methods which predict drug classes well will also do better at predicting disease indications for drugs.

Iskar *et al.* [14] presented the first quantitative evaluation of CMAP methods for identifying similar compounds. They used compound chemical similarity and Anatomical Therapeutic Chemical (ATC) classification as the true positives in their benchmark sets. Using the benchmark sets, Iskar *et al.* computed the early retrieval performance of their approach and showed that their proposed approach performs better than previous methods [15]. Early retrieval performance was measured using the partial area under the receiver operator characteristic (ROC) curve (AUC0.1) at false positive rate 0.1 (FPR = 0.1), which is important from a drug repositioning perspective as it is only practical to investigate a small number of top hypotheses.

Cheng *et al.* [16] also utilized the ATC classification as the benchmark to compare similarity metrics using two data processing methods: the batch DMSO control and the mean centering normalization. The results from these studies show that a simple eXtreme cosine (XCos; see Methods section) similarity metric outperforms the standard KS similarity metric; and the batch DMSO control preprocessing outperforms mean centering normalization preprocessing in terms of early retrieval accuracy. Another important finding from this study is that drug-drug relationships can be predicted more reliably when the corresponding compounds have large treatment effect on the treated cell lines. Cheng *et al.* [17] further extended the aforementioned work by evaluating various CMAP similarity metrics across different feature sizes by using only those compound profiles that have large treatment effect.

The current study is based on the conclusions drawn from the previous studies. Instead of predicting drug-drug relationships, we evaluate the CMAP performance in a more direct drug repurposing setting: predicting drug-disease relationships. We compiled a set of 890 true drug-disease pairs from two different sources (see Methods) as a benchmarking standard. The goal of this study is to evaluate CMAP methodology (focusing primarily on early retrieval performance) in a realistic setting using a large set of disease gene signatures that have reasonable overall predictive power.

## Methods

### Data sources and data processing

We downloaded the Connectivity Map (CMAP, build 02, [18]) data, which comprises 6,100 gene expression instances (treatment vs. vehicle control pairs) from primarily three human cultured cell lines (MCF7, PC3, and HL60) treated with 1,309 bioactive small chemical molecules at varying concentrations.

In our previous work [16], we evaluated two methods to preprocess probe level intensities - the mean centering method and the batch DMSO control method and found the batch DMSO control method appears to perform better than mean centering method. Based on this evaluation, we incorporated the batch DMSO control method in this study for the data preprocessing step.

Probe level data (CEL files) from CMAP were processed using Array Studio (Omicsoft Corporation, Research Triangle Park, NC, USA). Briefly, microarray datasets were grouped based on the cell line. For each microarray dataset, the probe set intensities were normalized using Robust Multi-array Average (RMA) procedure. Next, all scaled probe sets with values less than primary threshold values (set to 128) for all treatments and control samples were set to that threshold value (128). The intensity values for each probe set are then log2 transformed. Finally, the log2 intensities of each probe set from all vehicle control samples within the same batch and cell line are averaged and subtracted from the treatment sample to generate the corresponding treatment-to-control values. These treatment-to-control values are referred as expression values in this paper.

The preprocessing step resulted in 6,100 gene expression instances. Multiple instances for the same compound in a particular cell line were averaged to generate cell line specific compound profiles. There are 1,294, 1,182, and 1,078 compound profiles for MCF7, PC3, and HL60 cell lines, respectively.

### Compound expression profile filtering by expression signal strength

Cheng *et al.* [16] show that the majority of the compounds do not have large enough treatment effect on the cell lines to obtain reliable ATC group prediction. We follow the compound filtering procedure described by Cheng *et al.* [17] and use expression signal strength (ESS) to filter the cell line specific compound profiles. The ESS is defined as the sum of the absolute values of the log2 of the fold changes of the top and bottom N features of a gene expression profile (N is set to 50 in this study). The cell line specific threshold for compound profile filtering is defined as following.

$$ ESS\  Threshold= median\ ESS+2\times median\  absolute\  deviation $$

An example of ESS histogram of compound profiles and the ESS threshold is shown in Additional file 1: Figure S1. After the filtering step, we kept 297, 222, and 246 compound profiles for MCF7, PC3 and HL60, respectively.

**Figure 1:**
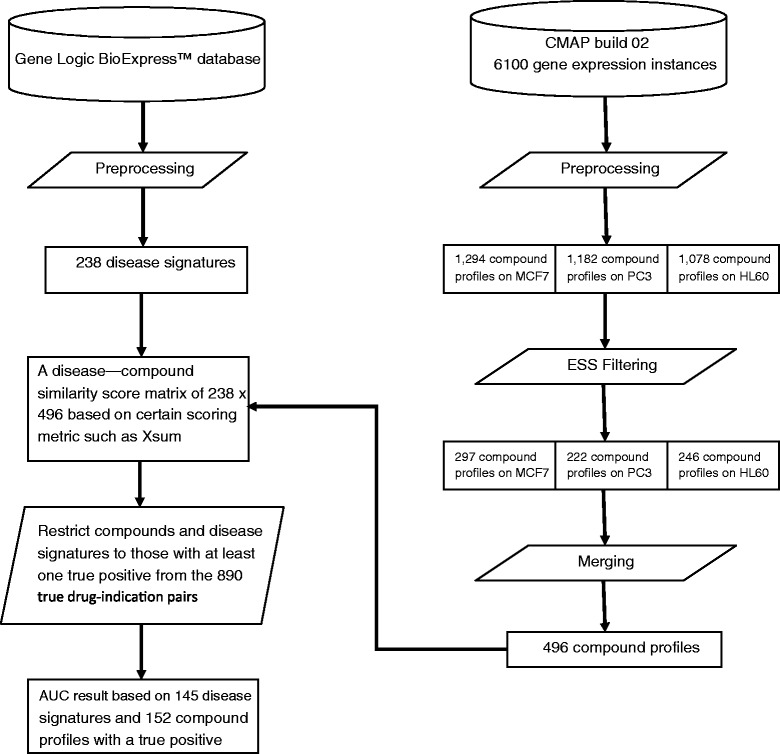
**Work flow of connectivity map performance evaluation.**

In the final step, we merged the cell-line-specific compound profiles into the compound level profiles by averaging the expression values across cell lines. This step resulted in 496 compound expression profiles, which were used to calculate different expression similarity metrics (see Additional file 1: Table S1). The overall work flow of our system is shown in Figure 1.

### Disease gene signature generation and evaluation

Gene expression data containing Affymetrix U133 GeneChip expression profiles (Affymetrix, Santa Clara, CA, USA; [19]) for over 13,000 samples (9,990 diseased; 3,400 normal) across 124 human tissues were retrieved from the Gene Logic BioExpress™ system [20]. The BioExpress System provides gene expression data and associated clinical and pathological information across a wide range of normal and diseased specimens across major disease areas including cardiovascular, oncology, central nervous system, inflammatory, and metabolic diseases. For each study conducted at Genelogic, the research protocol was approved by one or more institutional review boards, and all participating patients provided written informed consent consistent with the principles of the Declaration of Helsinki [20].

All data analyses, including preprocessing and normalization, and summarization of the AffyBatch probe level data, were carried out using R package ‘Bioconductor 2.9’ [21]. To generate an individual disease signature, samples were grouped by the tissue of origin, study ID and disease categorization. Only tissues with at least three normal and three disease samples were considered for further processing. A pairwise sample correlation was computed across all samples using the entire chip data. Then for each sample, an average correlation coefficient was generated based on correlation coefficients between the given sample and all other samples. All samples with an average correlation of more than five median absolute deviations (MAD) from the median of all sample correlations were deemed to be an outlier and removed from further consideration.

A total of 238 disease signatures from human clinical disease samples were generated. Each disease gene signature consists of 500 upregulated probe sets and 500 downregulated probe sets selected by fold change between disease samples and normal samples. Among the 238 diseases, 145 diseases have at least one true positive compound in our compiled benchmark standard and were further evaluated (see Additional file 1: Table S2).

### Compilation of true drug-indication relationships (benchmark standard)

We identified the established drug-indication relationships from two data sources: Pharmaprojects pipeline® [22] and FDA adverse event reporting system (FAERS). The Pharmaprojects pipeline® is a resource compiling worldwide drug pipeline data including their disease indications. Each Pharmaprojects disease term was also mapped to MeSH via manual curation. The disease names for the signatures used in our study (from GeneLogic) use SNOMED as a coding system. We mapped the GeneLogic disease terms to MeSH using UMLS [23]. The MeSH terms were used as a bridge to connect GeneLogic diseases with the Pharmaprojects drug indications.

Drug indication information from the FDA adverse event reporting system (FAERS) was downloaded from the FDA website [24], where the indication of a drug were linked to the drug name via the fields ISR and DRUG_SEQ The drug names in FAERS were mapped to pipeline® drug names via drug synonyms field in the pipeline® database. The MedDRA terms from the FAERS data were mapped to MeSH terms through manual curation. Only drug-disease relationships that were backed by at least 50 reports were incorporated in this study to insure there are sufficient use cases of the drug in certain disease indications. We combined the two drug-indication pairs and used them as the benchmark standard to perform the evaluation (see Additional file 1: Table S3).

### Pairwise similarity metrics

Different similarity metrics have been used to match gene signatures (profiles), such as Kolmogorov-Smirnov (KS) statistic [5] and the weighted signed statistics [25,26]. In this study, we focus on three metrics: XSum, XCos, and KS metric to predict drug-disease relationships. The focus of this paper is not to evaluate all metrics, but to show that at least one metric can work to find disease indications. These metrics are briefly described as follows.

*KS:* The initial CMAP approach utilized a non-parametric, rank-based Kolmogorov-Smirnov (KS) statistic defined as follows.

$$ UpInDisease=\mathrm{a}\ \mathrm{set}\ \mathrm{of}\ \mathrm{N}\ \mathrm{up}\hbox{-} \mathrm{regulated}\ \mathrm{features}\ \mathrm{from}\ \mathrm{disease}\ \mathrm{genomic}\ \mathrm{data} $$

$$ DownInDisease=\mathrm{a}\ \mathrm{set}\ \mathrm{of}\ \mathrm{N}\ \mathrm{down}\hbox{-} \mathrm{regulated}\ \mathrm{features}\ \mathrm{from}\ \mathrm{disease}\ \mathrm{genomic}\ \mathrm{data} $$

$$ K{S}_{up}=\mathrm{the}\ \mathrm{K}\mathrm{S}\ \mathrm{score}\ \mathrm{between}\; UpInDisease\;\mathrm{and}\ \mathrm{complete}\ \mathrm{compound}\ \mathrm{profile} $$

$$ K{S}_{down}=\mathrm{the}\ \mathrm{K}\mathrm{S}\ \mathrm{score}\ \mathrm{between}\; DownInDisease\ \mathrm{and}\ \mathrm{complete}\ \mathrm{compound}\ \mathrm{profile} $$

$$ IfK{S}_{up}\;\mathrm{and}\;K{S}_{down}\;\mathrm{have}\ \mathrm{different}\ \mathrm{signs}\ \mathrm{then}\; Connectivity\  score=K{S}_{up} - K{S}_{down}\; else\  Connectivity\  score=0 $$

For this study, N is set to 500 in all metrics.

*XSum:* The eXtreme Sum score is calculated as follows.

$$ UpInDisease\;\mathrm{and}\; DownInDisease\  are\  defined\ as\  above $$

$$ \begin{array}{l} ChangedByCompound=\mathrm{top}\ \mathrm{N}\ \mathrm{up}\hbox{-} \mathrm{regulated}\ \mathrm{and}\ \mathrm{N}\ \mathrm{down}\hbox{-} \mathrm{regulated}\ \mathrm{features}\ \mathrm{b}\mathrm{y}\ \\ {}\mathrm{fold}\ \mathrm{change}\ \mathrm{values}\ \mathrm{b}\mathrm{etween}\ \mathrm{compound}\ \mathrm{treated}\ \mathrm{samples}\ \mathrm{and}\ \mathrm{control}\ \mathrm{samples}\end{array} $$

$$ XUpInDisease= UpInDisease\cap ChangedByCompound $$

$$ XDownInDisease= DownInDisease\cap ChangedByCompound $$

$$ sum(XUpInDisease)=s\mathrm{u}\mathrm{m}\ \mathrm{of}\ \mathrm{compound}\ \mathrm{gene}\ \mathrm{expression}\ \mathrm{fold}\ \mathrm{change}\ \mathrm{values}\ \mathrm{in}\ \mathrm{the}\ \mathrm{set}\; XUpInDisease $$

$$ sum(XDownInDisease)=\mathrm{sum}\ \mathrm{of}\ \mathrm{compound}\ \mathrm{gene}\ \mathrm{expression}\ \mathrm{fold}\ \mathrm{change}\ \mathrm{values}\ \mathrm{in}\ \mathrm{the}\ \mathrm{set}\  XDownInDisease $$

$$ \mathrm{XSum}=\mathrm{sum}(XUpInDisease)-\mathrm{sum}(XDownInDisease) $$

XSum has some similarity with KS - they both use two gene lists (*UpInDisease* and *DownInDisease*) to query compound profiles and then measure the difference between the two corresponding scores. Instead of using KS score to check whether the queried genes are concentrated at the top or bottom of the sorted compound profile, XSum simply sums up the compound gene expression values of the queried genes.

*XCos:* The eXtreme cosine similarity score is calculated as follows.

$$ XSet=\left( UpInDisease\cup DownInDisease\right)\ \cap\ (ChangedByCompound) $$

$$ XCos=\mathrm{the}\ \mathrm{cosine}\ \mathrm{similarity}\ \mathrm{of}\ \mathrm{the}\ \mathrm{disease}\ \mathrm{fold}\ \mathrm{change}\ \mathrm{vector}\ \mathrm{and}\ \mathrm{compound}\ \mathrm{profile}\ \mathrm{vector}\ \mathrm{using}\ \mathrm{features}\ \mathrm{of}\  XSet $$

### AUCs and permutation *P* values

We use multiple areas under the curve (AUC) of the receiver operator characteristic (ROC) measures to evaluate CMAP performance, focusing on early retrieval performance where the false positive rate is low: FPR = 0.01 and FPR = 0.1 (specificity 0.99 and 0.9, respectively). The rationale for using this approach is we desire high specificity because we can only pursue a limited number of drug-disease indication hypotheses in a drug repositioning exercise. We calculate two types of AUC numbers: *AvgAUC* is the AUC averaged (unweighted) across all disease signatures, and *OneAUC* is calculated by combining the prediction scores across all the disease signatures and then calculating a single overall AUC.

To determine the statistical significance of the AUC results, we calculate non-parametric *P* values by performing 10,000 runs with random permutations of the 890 disease mappings of the compounds.

## Results

In total, 145 of the 238 diseases have at least one true positive compound in our compiled benchmark standard and thus can be evaluated (see Figure 1 and Additional file 1: Table S2). Likewise, 152 of the 496 compounds had at least one signature for an indicated disease. The experimental result using compounds filtered using Expression Signal Strength (ESS) is shown in Tables 1 and 2. The AUCs for XSum are significantly different than random at false positive rates of 0.01 and 0.1, as well as across the entire ROC. XSum also enriches 3.8-fold for positives at a false positive rate of 0.01. XCos is only statistically significant across the entire ROC, while KS does not show significance. To demonstrate the effectiveness of the compound filtering process, we reported the experimental result on all unfiltered compounds in Additional file 1: Table S4, where none of the AUC measure is statistically significant.

**Table 1:** **Partial AUC performance and random permutation** ***P*** **value for three metrics using 152 filtered compound profiles**

**Method**	**FPR = 0.01 (Specificity = 0.99)**		**FPR = 0.1 (Specificity = 0.9)**		
	**Fold enrichment**	**OneAUC0.01**	**Fold enrichment**	**OneAUC0.1**	**AvgAUC0.1**
KS	0.7	3.17e-5 (*P* = 0.94)	0.9	0.005 (*P* = 0.81)	0.003 (*P* = 0.90)
XSum	**3.8**	**2.17e-4 (** ***P*** **= 0.0035)**	**1.4**	**0.0086 (** ***P*** **= 0.014)**	**0.011 (** ***P*** **= 0.035)**
XCos	2.3	1.82e-4 (*P* = 0.056)	1.2	0.006 (*P* = 0.16)	0.006 (*P* = 0.37)

AvgAUC0.01 is not calculated because of limited sample size. Fold enrichment is calculated as the ratio between true positive rate and false positive rate.

**Table 2:** **Complete AUC performance and random permutation** ***P*** **value for three metrics using 152 filtered compound profiles**

**Method**	**AvgAUC**	**OneAUC**
KS	0.48 (*P* = 0.75)	0.48 (*P* = 0.83)
XSum	**0.56 (** ***P*** **= 0.031)**	**0.54 (** ***P*** **= 0.025)**
XCos	**0.56 (** ***P*** **= 0.049)**	**0.54 (** ***P*** **= 0.039)**

The AUC performance of the three different metrics in predicting drug-disease relationships is consistent with our earlier finding [16], which is based on predicting drug-drug relationships. XSum is a robust metric for CMAP as it has statistically significant *P* values in all five AUC measures, which demonstrates that the CMAP approach can achieve better than random performance in predicting drug-disease relationships. The top 20 disease-drug pairs ranked by XSum are shown in Additional file 1: Table S5.

All partial and full ROC curves using XSum as the classifier are plotted in Additional file 1: Figure S2.

We also grouped the disease signatures into two categories: neoplastic (65 disease signatures) and non-neoplastic (80 disease signatures) and evaluated the AUC performance of XSum for each category (see Table 3). The result shows that XSum gives better performance for neoplastic signatures, especially in term of early retrieval performance (OneAUC0.1 and OneAUC0.01), where the neoplastic diseases achieve much significant *P* values (0.006 and 0.001, respectively). In fact, the performance for non-neoplastic disease does not achieve significance; this suggests that perhaps better samples or methods might be needed for the non-neoplastic diseases.

**Table 3:** **AUC performance and random permutation** ***P*** **value for XSum using 152 filtered compound profiles on neoplastic and non-neoplastic disease signatures**

**Disease signature group**	**AvgAUC**	**OneAUC**	**OneAUC0.1**	**OneAUC0.01**
Neoplastic	**0.61 (** ***P*** **= 0.05)**	0.6 (*P* = 0.06)	**0.016 (** ***P*** **= 0.006)**	**6e-4 (** ***P*** **= 0.001)**
Non-neoplastic	0.52 (*P* = 0.16)	0.53 (*P* = 0.09)	0.0057 (*P* = 0.44)	3e-5 (*P* = 0.78)

To illustrate how the quality of disease gene signature can affect the CMAP performance, we compared a validated breast cancer prognostic signature [27] with one of the breast cancer gene signatures in our 145 disease signature list, by querying the 496 filtered compounds using XSum (see Table 4). For the validated breast cancer prognosis signature, the top three compounds are anti-cancer drugs and fifth ranked compound MG-262 is also known for its anti-cancer effect; while for the Gene Logic signature, none of the five compounds are anti-cancer drugs. It suggests that the CMAP performance may be greatly improved by using higher quality disease gene signatures. However, this is just one case study. In future work, it is worth evaluating higher quality signatures from multiple diseases and re-assessing their CMAP performance.

**Table 4:** **Top five compounds matched to a breast cancer gene signature from Gene Logic (Breast: Intraductal carcinoma: Primary malignant neoplasm of female breast) and top five compounds matched to a validated breast cancer prognostic gene signature**

**Top 5 ranked compounds from a validated breast cancer prognostic signature**	**Top 5 ranked compounds from a Gene Logic breast cancer signature**
**Irinotecan**	Mesalazine
**Methotrexate**	Ambroxol
**Etoposide**	Alclometasone
5109870	Verteporfin
*MG-262*	Beclometasone

XSum is used for matching. Known anti-cancer drugs are in bold.

We also used the same breast cancer prognostic signature to query the standard CMAP tool (build 02) from Broad Institute (available online at [18]). The results shown in Table 5 are consistent with our findings indicating XSum is more effective than KS in early retrieval performance.

**Table 5:** **Top five compounds matched to a validated breast cancer prognostic gene signature using our approach and the standard CMAP approach**

**Top 5 ranked compounds based on XSum score**	**Top 5 ranked compounds (by** ***P*** **value) from CMAP online tool**
**Irinotecan**	*Chlorpromazine*
**Methotrexate**	*Resveratrol*
**Etoposide**	*Luteolin*
5109870	Morantel
*MG-262*	*Antimycin A*

Known anti-cancer drugs are in bold.

## Discussion

We systematically evaluated multiple CMAP methodologies by assessing their performance on 152 drug profiles across a curated dataset consisting of 145 disease gene signatures. This large comprehensive study can serve as a benchmark to assess any new methodologies in the future. The results from this study show that by using a simple scoring algorithm called *eXtreme Sum* (XSum) we were able to achieve a significantly improved metric with the AUC around 0.57 and having a significant permutation *P* value (*P* = 0.03). In an earlier study and on a smaller set of the drugs and diseases (19 disease signatures and 69 drugs), the best metric obtained had an AUC of 0.45. The result of this study confirms two factors identified in our earlier work [16,17] which are crucial to the successful application of CMAP - an effective similarity metric such as XSum and a proper procedure for filtering the compound data. Using XSum and the compound filtering procedure, we show CMAP can give better than random performance on a relatively large number of gene signatures, which were generated from a single source using a simplistic protocol. XSum has a promising early retrieval performance when FPR = 0.01, which is most relevant for CMAP-based drug repositioning, as we normally can only afford to experimentally investigate a small number of top hypotheses. However, the overall AUC performance is rather weak. There are several possible reasons. First, not all disease gene signatures are of high quality. Some of the disease signatures may have random or worse than random performance. Moreover, many diseases may not be represented accurately by the transcriptional response in the current signatures. Second, the drug-disease benchmark standard may not necessarily capture all known drug-disease association as it may contain both false negatives (drugs could treat the disease but have not been tested yet), and false positives (drugs that may only provide symptomatic relief and thus may not be able to reversibly match to the disease signature). Third, we did not try to optimize the CMAP performance, as that process can be prone to over fitting and bias. Rather, our goal was to evaluate CMAP in a simple and easy-to-follow procedure. Future work may include fine tuning the parameters of XSum, such as the number of features to be included in disease signature and compound profiles. Last but not least, current compound cellular profiles are limited to compound treatment of three cancer cell lines, which is possibly one of the reasons that neoplastic disease signatures perform better than non-neoplastic disease signatures (see Table 3). The availability of more cell types, drug treatments, gene knockout data, and gene over expression data from the LINCS project should enable much larger and more thorough analysis in the near future [28].

## Conclusions

Connectivity map can significantly enrich true positive drug-indication pairs given an effective matching algorithm especially for neoplastic diseases. In combination with the numerous anecdotal examples of successful experimental validation of CMAP hypotheses, this increases confidence in the use of CMAP for drug repurposing. With better methods and stronger validation datasets, it is our belief that CMAP will prove to be an effective method to repurpose drugs across a broad range of diseases.

## Additional file

Additional file 1: Figure S1 Expression signal strength of 1,294 compound expression profiles on MCF7 cell line. Top 290 profiles are kept (ESS threshold = 44). (a) ESS value sorted from large to small; (b) Histogram of ESS value. **Figure S2.** ROC curves of classifying drugs across different disease signatures using Xsum for the FPR = 0.01 (A) and FPR = 0.1 (B) and the full ROC curve (C). **Table S1.** The list of 496 compounds that passed the expression signal strength filtering. **Table S2.** The list of 145 disease signatures and their XSum performance (sorted by lower bound of AUC with 95% confidence interval). **Table S3.** Drug-indication relationships (benchmark standard). **Table S4.** AUC performance and random permutation *P* value for three metrics using all (unfiltered) compound profiles. **Table S5.** Top 20 disease-drug pairs by XSum.

## References

[1] Dudley JT, Deshpande T, Butte AJ (2011). Exploiting drug-disease relationships for computational drug repositioning. Brief Bioinform.

[2] Suthram S, Dudley JT, Chiang AP, Chen R, Hastie TJ, Butte AJ (2010). Network-based elucidation of human disease similarities reveals common functional modules enriched for pluripotent drug targets. PLoS Comput Biol.

[3] Hu G, Agarwal P (2009). Human disease-drug network based on genomic expression profiles. PLoS One.

[4] Sirota M, Dudley JT, Kim J, Chiang AP, Morgan AA, Sweet-Cordero A, Sage J, Butte AJ (2011). Discovery and preclinical validation of drug indications using compendia of public gene expression data. Sci Transl Med.

[5] Lamb J, Crawford ED, Peck D, Modell JW, Blat IC, Wrobel MJ, Lerner J, Brunet JP, Subramanian A, Ross KN, Reich M, Hieronymus H, Wei G, Armstrong SA, Haggarty SJ, Clemons PA, Wei R, Carr SA, Lander ES, Golub TR (2006). The Connectivity Map: using gene-expression signatures to connect small molecules, genes, and disease. Science.

[6] Qu XA, Rajpal DK (2012). Applications of Connectivity Map in Drug Discovery and Development. Drug Discov Today.

[7] Chang M, Smith S, Thorpe A, Barratt MJ, Karim F (2010). Evaluation of phenoxybenzamine in the CFA model of pain following gene expression studies and connectivity mapping. Mol Pain.

[8] Claerhout S, Lim JY, Choi W, Park YY, Kim K, Kim SB, Lee JS, Mills GB, Cho JY (2011). Gene expression signature analysis identifies vorinostat as a candidate therapy for gastric cancer. PLoS One.

[9] Dudley JT, Sirota M, Shenoy M, Pai RK, Roedder S, Chiang AP, Morgan AA, Sarwal MM, Pasricha PJ, Butte AJ (2011). Computational repositioning of the anticonvulsant topiramate for inflammatory bowel disease. Sci Transl Med.

[10] Ishimatsu-Tsuji Y, Soma T, Kishimoto J (2010). Identification of novel hair-growth inducers by means of connectivity mapping. FASEB J.

[11] Kunkel SD, Suneja M, Ebert SM, Bongers KS, Fox DK, Malmberg SE, Alipour F, Shields RK, Adams CM (2011). mRNA expression signatures of human skeletal muscle atrophy identify a natural compound that increases muscle mass. Cell Metab.

[12] Jin L, Tu J, Jia J, An W, Tan H, Cui Q, Li Z (2014). Drug-repurposing identified the combination of Trolox C and Cytisine for the treatment of type 2 diabetes. J Transl Med.

[13] Gottlieb A, Stein GY, Ruppin E, Sharan R (2011). PREDICT: a method for inferring novel drug indications with application to personalized medicine. Mol Syst Biol.

[14] Iskar M, Campillos M, Kuhn M, Jensen LJ, van Noort V, Bork P (2010). Drug-induced regulation of target expression. PLoS Comput Biol.

[15] Iorio F, Bosotti R, Scacheri E, Belcastro V, Mithbaokar P, Ferriero R, Murino L, Tagliaferri R, Brunetti-Pierri N, Isacchi A, di Bernardo D (2010). Discovery of drug mode of action and drug repositioning from transcriptional responses. Proc Natl Acad Sci U S A.

[16] Cheng J, Xie Q, Kumar V, Hurle M, Freudenberg JM, Yang L, Agarwal P (2013). Evaluation of analytical methods for connectivity map data. Pac Symp Biocomput.

[17] Cheng J, Yang L, Li G, Hu X, Kim S, Ressom H, Hughes M, Liu B, McLachlan G, Liebman M, Sun H (2013). Comparing gene expression similarity metrics for connectivity map. Proceedings of IEEE International Conference on Bioinformatics and Biomedicine: 18–21 Dec. 2013, Shanghai, China.

[18] **The Connectivity map 02.** [http://www.broadinstitute.org/cmap/]

[19] **Affymetrix.** [http://www.affymetrix.com]

[20] **Genelogic.** [http://www.ocimumbio.com/bioexpress-system/]

[21] Gentleman RC, Carey VJ, Bates DM, Bolstad B, Dettling M, Dudoit S, Ellis B, Gautier L, Ge Y, Gentry J, Hornik K, Hothorn T, Huber W, Iacus S, Irizarry R, Leisch F, Li C, Maechler M, Rossini AJ, Sawitzki G, Smith C, Smyth G, Tierney L, Yang JY, Zhang J (2004). Bioconductor: open software development for computational biology and bioinformatics. Genome Biol.

[22] **Pharmaprojects.** [http://www.citeline.com/products/pharmaprojects/]

[23] **Unified Medical Language System.** [http://www.nlm.nih.gov/research/umls/]

[24] **FDA Adverse Event Reporting System (FAERS).** [http://www.fda.gov/Drugs/GuidanceComplianceRegulatoryInformation/Surveillance/AdverseDrugEffects/]

[25] Zhang SD, Gant TW (2008). A simple and robust method for connecting small-molecule drugs using gene-expression signatures. BMC Bioinformatics.

[26] Pacini C, Iorio F, Gonçalves E, Iskar M, Klabunde T, Bork P, Saez-Rodriguez J (2013). DvD: An R/Cytoscape pipeline for drug repurposing using public repositories of gene expression data. Bioinformatics.

[27] Cheng J, Greshock J, Shi L, Zheng S, Menius A, Lee K (2013). Good practice guidelines for biomarker discovery from array data: a case study for breast cancer prognosis. BMC Syst Biol.

[28] Vempati UD, Chung C, Mader C, Koleti A, Datar N, Vidovic D, Wrobel D, Erickson S, Muhlich JL, Berriz G, Benes CH, Subramanian A, Pillai A, Shamu CE, Schürer SC (2014). Metadata standard and data exchange specifications to describe, model, and integrate complex and diverse high-throughput screening data from the Library of Integrated Network-based Cellular Signatures (LINCS). J Biomol Screen.

