# Supplementary material for: Systematic evaluation of connectivity map for disease indications
Source: Genome Med. 2014 Dec 2;6:95. doi: 10.1186/s13073-014-0095-1 (PMC4278345; doi:10.1186/s13073-014-0095-1)
Supplement: Additional file 1: Figure S1 — Expression signal strength of 1,294 compound expression profiles on MCF7 cell line. Top 290 profiles are kept (ESS threshold = 44). (a) ESS value sorted from large to small; (b) Histogram of ESS value. Figure S2. ROC curves of classifying drugs across different disease signatures using Xsum for the FPR = 0.01 (A) and FPR = 0.1 (B) and the full ROC curve (C). Table S1. The list of 496 compounds that passed the expression signal strength filtering. Table S2. The list of 145 disease signatures and their XSum performance (sorted by lower bound of AUC with 95% confidence interval). Table S3. Drug-indication relationships (benchmark standard). Table S4. AUC performance and random permutation P value for three metrics using all (unfiltered) compound profiles. Table S5. Top 20 disease-drug pairs by XSum. [file 13073_2014_95_MOESM1_ESM.docx]

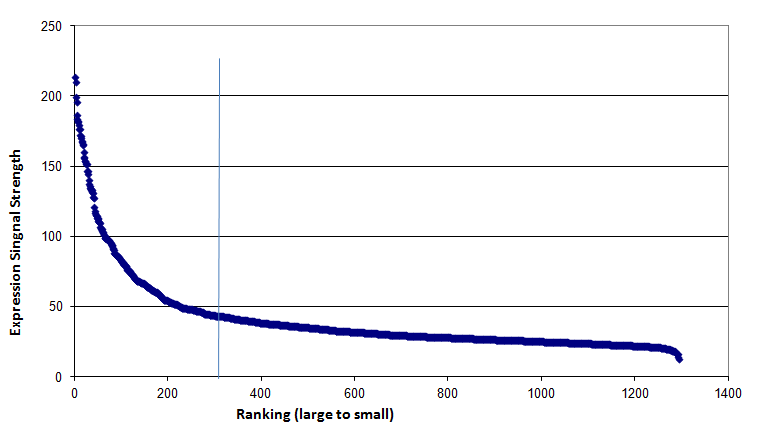


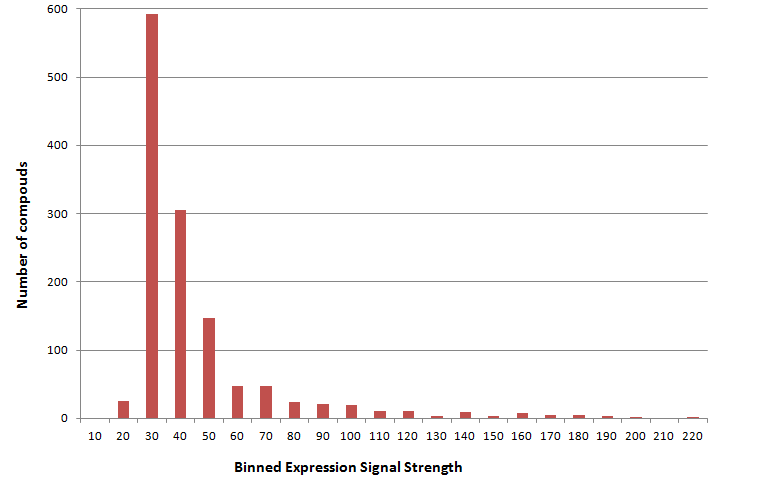


Figure S1. Expression signal strength of 1,294 compound expression profiles on MCF7 cell line. Top 290 profiles are kept (ESS threshold =44). (a) ESS value sorted from large to small; (b) Histogram of ESS value.


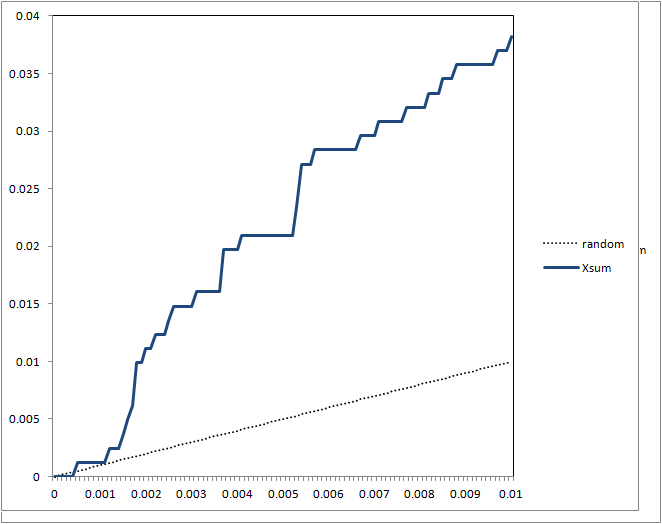

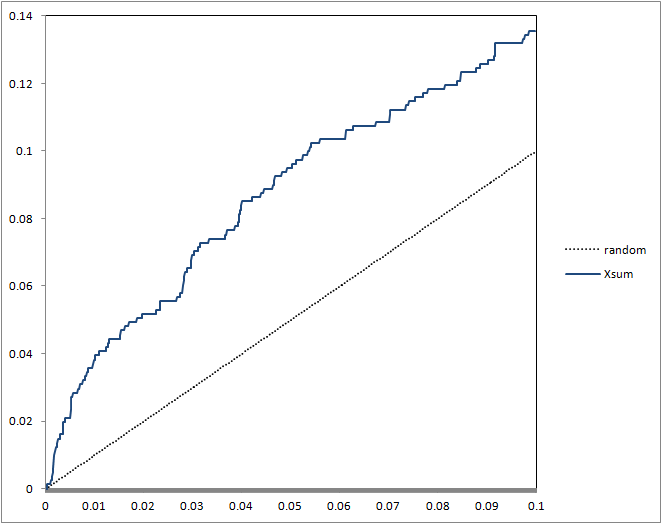


B

A

C

Figure S2. ROC curves of classifying drugs across different disease signatures using Xsum for the FPR = 0.01 (A) and FPR = 0.1 (B) and the full ROC curve (C).

Table S1: The list of 496 compounds that passed the expression signal strength filtering

| 5109870 |
| --- |
| 5114445 |
| 5155877 |
| 5182598 |
| 5186223 |
| 5194442 |
| 5213008 |
| 5224221 |
| 5253409 |
| 5707885 |
| (-)-isoprenaline |
| (+)-chelidonine |
| (+)-isoprenaline |
| 0173570-0000 |
| 0175029-0000 |
| 0179445-0000 |
| 0225151-0000 |
| 0297417-0002B |
| 0316684-0000 |
| 0317956-0000 |
| 1,4-chrysenequinone |
| 1,5-isoquinolinediol |
| 11-deoxy-16,16-dimethylprostaglandin E2 |
| 15-delta prostaglandin J2 |
| 16,16-dimethylprostaglandin E2 |
| 16-phenyltetranorprostaglandin E2 |
| 2-deoxy-D-glucose |
| 3-aminobenzamide |
| 4,5-dianilinophthalimide |
| 6-azathymine |
| 6-bromoindirubin-3'-oxime |
| 8-azaguanine |
| abamectin |
| acacetin |
| acepromazine |
| adenosine phosphate |
| AG-012559 |
| AG-028671 |
| AH-23848 |
| ajmaline |
| albendazole |
| alclometasone |
| alexidine |
| alfadolone |
| alimemazine |
| alpha-ergocryptine |
| alprostadil |
| alsterpaullone |
| alvespimycin |
| amantadine |
| ambroxol |
| amikacin |
| ampyrone |
| anisomycin |
| antimycin A |
| apigenin |
| AR-A014418 |
| arachidonyltrifluoromethane |
| astemizole |
| atovaquone |
| azacitidine |
| azacyclonol |
| azapropazone |
| azathioprine |
| aztreonam |
| baclofen |
| BAS-012416453 |
| BCB000038 |
| BCB000039 |
| BCB000040 |
| beclometasone |
| benfluorex |
| benfotiamine |
| benperidol |
| benserazide |
| benzbromarone |
| benzethonium chloride |
| benzocaine |
| benzylpenicillin |
| bepridil |
| berberine |
| beta-escin |
| betamethasone |
| betazole |
| betulin |
| betulinic acid |
| biotin |
| bisacodyl |
| bisoprolol |
| bromocriptine |
| budesonide |
| buflomedil |
| butein |
| BW-B70C |
| C-75 |
| caffeic acid |
| calmidazolium |
| camptothecin |
| canavanine |
| cantharidin |
| captopril |
| carbachol |
| carmustine |
| CAY-10397 |
| cefadroxil |
| cefalexin |
| cefalotin |
| cefepime |
| ceforanide |
| cefotiam |
| celastrol |
| cephaeline |
| chlorhexidine |
| chloropyramine |
| chlorpromazine |
| chlorpropamide |
| chlorprothixene |
| chlortetracycline |
| chlorzoxazone |
| chrysin |
| cicloheximide |
| ciclopirox |
| ciclosporin |
| cisapride |
| clemastine |
| clenbuterol |
| clindamycin |
| clioquinol |
| clobetasol |
| clofibrate |
| clofilium tosylate |
| clomifene |
| clomipramine |
| clonidine |
| clopamide |
| cloperastine |
| clotrimazole |
| co-dergocrine mesilate |
| colchicine |
| colforsin |
| corbadrine |
| corticosterone |
| CP-319743 |
| CP-645525-01 |
| CP-690334-01 |
| CP-863187 |
| crotamiton |
| cyclopenthiazide |
| cytochalasin B |
| danazol |
| daunorubicin |
| debrisoquine |
| deferoxamine |
| demecolcine |
| deptropine |
| dequalinium chloride |
| desipramine |
| dexamethasone |
| dexibuprofen |
| dexverapamil |
| diclofenamide |
| diethylstilbestrol |
| diflorasone |
| digitoxigenin |
| digoxigenin |
| digoxin |
| dihydroergocristine |
| dihydroergotamine |
| dilazep |
| diltiazem |
| dimethyloxalylglycine |
| dinoprost |
| dinoprostone |
| dipivefrine |
| dipyridamole |
| dirithromycin |
| disopyramide |
| disulfiram |
| dizocilpine |
| dobutamine |
| docosahexaenoic acid ethyl ester |
| domperidone |
| dorzolamide |
| doxorubicin |
| dydrogesterone |
| ebselen |
| econazole |
| ellipticine |
| emetine |
| epivincamine |
| erastin |
| estradiol |
| estrone |
| etacrynic acid |
| ethaverine |
| ethosuximide |
| ethotoin |
| etifenin |
| etilefrine |
| etoposide |
| etynodiol |
| exemestane |
| F0447-0125 |
| fasudil |
| felodipine |
| fenbendazole |
| fendiline |
| fenoterol |
| fisetin |
| flucytosine |
| fludroxycortide |
| flumequine |
| flumetasone |
| flunisolide |
| flunixin |
| fluorometholone |
| flupentixol |
| fluphenazine |
| fluspirilene |
| flutamide |
| fluticasone |
| fluvastatin |
| fulvestrant |
| furazolidone |
| gabexate |
| gefitinib |
| geldanamycin |
| genistein |
| glibenclamide |
| glycocholic acid |
| Gly-His-Lys |
| gossypol |
| GW-8510 |
| H-7 |
| H-89 |
| halcinonide |
| harmaline |
| harmine |
| harmol |
| HC toxin |
| helveticoside |
| hesperidin |
| hexamethonium bromide |
| hexetidine |
| hexylcaine |
| HNMPA-(AM)3 |
| homochlorcyclizine |
| hycanthone |
| hydrocortisone |
| hydrocotarnine |
| hyoscyamine |
| iloprost |
| imatinib |
| imipramine |
| indometacin |
| iohexol |
| ionomycin |
| iopanoic acid |
| ipratropium bromide |
| irinotecan |
| isoetarine |
| isoflupredone |
| isotretinoin |
| isoxsuprine |
| ivermectin |
| kaempferol |
| ketanserin |
| ketoconazole |
| kinetin |
| labetalol |
| lanatoside C |
| lasalocid |
| latamoxef |
| laudanosine |
| letrozole |
| levonorgestrel |
| lithocholic acid |
| lobeline |
| lomustine |
| loperamide |
| loxapine |
| luteolin |
| LY-294002 |
| lycorine |
| maprotiline |
| mebendazole |
| meclofenoxate |
| medrysone |
| mefloquine |
| megestrol |
| meglumine |
| menadione |
| mepacrine |
| mephentermine |
| mercaptopurine |
| mesalazine |
| mestranol |
| meteneprost |
| metergoline |
| methapyrilene |
| methotrexate |
| methylbenzethonium chloride |
| methylergometrine |
| methylprednisolone |
| metitepine |
| metixene |
| metoclopramide |
| metoprolol |
| metronidazole |
| MG-132 |
| MG-262 |
| miconazole |
| midecamycin |
| mifepristone |
| minocycline |
| mitoxantrone |
| mometasone |
| monastrol |
| monensin |
| monobenzone |
| monorden |
| MS-275 |
| mycophenolic acid |
| myricetin |
| naftidrofuryl |
| naftifine |
| napelline |
| naringin |
| neostigmine bromide |
| niclosamide |
| nicotinic acid |
| nifedipine |
| nifuroxazide |
| nilutamide |
| nimodipine |
| nitrofural |
| nocodazole |
| norcyclobenzaprine |
| nortriptyline |
| nystatin |
| omeprazole |
| orciprenaline |
| ouabain |
| oxamic acid |
| oxaprozin |
| oxolinic acid |
| oxyphenbutazone |
| paclitaxel |
| papaverine |
| pararosaniline |
| parthenolide |
| pempidine |
| pentamidine |
| pentetrazol |
| pergolide |
| perhexiline |
| perphenazine |
| PF-00539745-00 |
| PF-00562151-00 |
| PF-00875133-00 |
| PHA-00665752 |
| PHA-00767505E |
| PHA-00851261E |
| phenanthridinone |
| phenoxybenzamine |
| picrotoxinin |
| pimozide |
| pioglitazone |
| pipemidic acid |
| piperacetazine |
| piperlongumine |
| piribedil |
| pivampicillin |
| pizotifen |
| PNU-0230031 |
| PNU-0251126 |
| PNU-0293363 |
| podophyllotoxin |
| prednisolone |
| pregnenolone |
| prenylamine |
| Prestwick-1080 |
| Prestwick-983 |
| primaquine |
| primidone |
| proadifen |
| prochlorperazine |
| profenamine |
| progesterone |
| promethazine |
| propantheline bromide |
| proscillaridin |
| protriptyline |
| puromycin |
| pyrantel |
| pyrimethamine |
| pyrithyldione |
| pyrvinium |
| quercetin |
| quinisocaine |
| quinostatin |
| quinpirole |
| raloxifene |
| R-atenolol |
| rescinnamine |
| reserpine |
| resveratrol |
| ribavirin |
| rifabutin |
| rimexolone |
| ritodrine |
| ronidazole |
| rosiglitazone |
| rotenone |
| rottlerin |
| salbutamol |
| sanguinarine |
| saquinavir |
| SC-560 |
| scopolamine |
| scoulerine |
| scriptaid |
| securinine |
| semustine |
| seneciphylline |
| simvastatin |
| sirolimus |
| skimmianine |
| sodium phenylbutyrate |
| solanine |
| spiperone |
| staurosporine |
| STOCK1N-28457 |
| STOCK1N-35215 |
| STOCK1N-35696 |
| strophanthidin |
| succinylsulfathiazole |
| sulconazole |
| sulfadimidine |
| sulfapyridine |
| sulfasalazine |
| sulindac |
| sulindac sulfide |
| sulmazole |
| suloctidil |
| sulpiride |
| syrosingopine |
| tacrine |
| tamoxifen |
| tanespimycin |
| terbutaline |
| terfenadine |
| tetrandrine |
| tetryzoline |
| thapsigargin |
| thioguanosine |
| thioridazine |
| thiostrepton |
| ticarcillin |
| ticlopidine |
| tioguanine |
| tolazoline |
| tolbutamide |
| tolmetin |
| tolnaftate |
| tomelukast |
| tonzonium bromide |
| tracazolate |
| tretinoin |
| trichostatin A |
| trifluoperazine |
| triflupromazine |
| trifluridine |
| trihexyphenidyl |
| trimipramine |
| triprolidine |
| troglitazone |
| TTNPB |
| tyrphostin AG-1478 |
| tyrphostin AG-825 |
| urapidil |
| valinomycin |
| valproic acid |
| vanoxerine |
| verapamil |
| verteporfin |
| vidarabine |
| vinblastine |
| vinburnine |
| vorinostat |
| withaferin A |
| wortmannin |
| zalcitabine |
| zardaverine |
| zidovudine |
| zuclopenthixol |

Table S2: The list of 145 disease signatures and their XSum performance (sorted by lower bound of AUC with 95% confidence interval)

| **Disease signature (***Tissue:Disease* or *Tissue:Disease:Disease subtype)* | Number of disease samples | Number of known positive compound(s) | Area under ROC curve | Std. Error of AUC |
| --- | --- | --- | --- | --- |
| Rectum:Adenocarcinoma:Primary malignant neoplasm of rectum | 52 | 1 | 0.99 | 0.057 |
| Rectum:Mucinous adenocarcinoma:Primary malignant neoplasm of rectum | 31 | 1 | 0.99 | 0.057 |
| Right atrium:Primary hypertrophic cardiomyopathy:Hypertrophy | 30 | 1 | 0.97 | 0.113 |
| Left atrium:Primary hypertrophic cardiomyopathy:Hypertrophy | 31 | 1 | 0.96 | 0.137 |
| Cervix:Squamous cell carcinoma:Primary malignant neoplasm of uterine cervix | 43 | 2 | 0.91 | 0.140 |
| Ascending colon:Acute and chronic inflammation | 36 | 15 | 0.69 | 0.079 |
| White blood cell:Rheumatoid arthritis | 61 | 17 | 0.67 | 0.075 |
| Lung:Adenocarcinoma:Primary malignant neoplasm of lung | 150 | 8 | 0.73 | 0.104 |
| Lung:Adenosquamous carcinoma:Primary malignant neoplasm of lung | 111 | 8 | 0.72 | 0.104 |
| Ascending colon:Adenocarcinoma:Primary malignant neoplasm of colon | 55 | 2 | 0.85 | 0.171 |
| Soft tissues:Liposarcoma | 7 | 1 | 0.91 | 0.201 |
| Soft tissues:Squamous cell carcinoma:Secondary malignant neoplasm of connective and other soft tissues | 10 | 2 | 0.84 | 0.175 |
| White blood cell:T-cell acute lymphoblastic leukemia | 33 | 7 | 0.72 | 0.112 |
| Liver:Adenoma:Benign neoplasm of liver | 35 | 3 | 0.80 | 0.155 |
| Lung:Carcinoma:Primary malignant neoplasm of lung | 117 | 8 | 0.69 | 0.106 |
| Liver:Steatosis:Fatty liver | 35 | 2 | 0.83 | 0.179 |
| Ovary:Mullerian mixed tumor:Primary malignant neoplasm of ovary | 69 | 3 | 0.79 | 0.158 |
| Bladder:Transitional cell carcinoma:Primary malignant neoplasm of bladder | 19 | 2 | 0.83 | 0.181 |
| Ileum:Acute and chronic inflammation:Crohns disease | 28 | 10 | 0.66 | 0.097 |
| Breast:Intraductal carcinoma:Primary malignant neoplasm of female breast | 35 | 13 | 0.64 | 0.086 |
| Endometrium:Hyperplasia:Endometrial hyperplasia | 15 | 3 | 0.78 | 0.160 |
| Skin:Chronic inflammation:Psoriasis | 82 | 19 | 0.60 | 0.073 |
| White blood cell:Ulcerative colitis | 35 | 7 | 0.67 | 0.114 |
| Soft tissues:Papillary serous adenocarcinoma:Secondary malignant neoplasm of connective and other soft tissues | 9 | 2 | 0.81 | 0.185 |
| Soft tissues:Adenocarcinoma:Secondary malignant neoplasm of connective and other soft tissues | 10 | 2 | 0.81 | 0.188 |
| Skeletal muscle:Diabetes mellitus | 34 | 7 | 0.66 | 0.115 |
| Blood:Coronary artery disease | 54 | 25 | 0.56 | 0.064 |
| Colon epithelial cell:Ulcerative colitis | 18 | 7 | 0.66 | 0.115 |
| Skin:Mycosis fungoides:Malignant lymphoma of extranodal AND/OR solid organ site | 57 | 3 | 0.75 | 0.165 |
| White blood cell:Osteoarthritis | 33 | 8 | 0.64 | 0.108 |
| Lung:Large cell carcinoma:Primary malignant neoplasm of lung | 114 | 8 | 0.63 | 0.108 |
| Breast:Infiltrating lobular carcinoma:Primary malignant neoplasm of female breast | 44 | 13 | 0.59 | 0.086 |
| Left ventricle:Fibrosis:Coronary artery disease | 24 | 25 | 0.53 | 0.064 |
| White blood cell:Crohns disease | 36 | 10 | 0.59 | 0.098 |
| Left ventricle:Recent infarct:Coronary artery disease | 48 | 25 | 0.52 | 0.064 |
| Lung:Squamous cell carcinoma:Primary malignant neoplasm of lung | 150 | 8 | 0.61 | 0.109 |
| Right ventricle:Primary hypertrophic cardiomyopathy:Hypertrophy | 39 | 1 | 0.86 | 0.236 |
| Blood:Psoriasis | 80 | 19 | 0.53 | 0.072 |
| Cecum:Adenocarcinoma:Primary malignant neoplasm of colon | 36 | 2 | 0.77 | 0.197 |
| Lung Epithelial cell:Primary malignant neoplasm of lung | 24 | 8 | 0.60 | 0.109 |
| Blood:Asthma | 48 | 17 | 0.52 | 0.075 |
| Descending colon:Adenocarcinoma:Primary malignant neoplasm of colon | 15 | 2 | 0.76 | 0.199 |
| Left ventricle:Old infarct:Coronary artery disease | 107 | 25 | 0.50 | 0.063 |
| bronchial brushings:Chronic obstructive lung disease | 23 | 11 | 0.55 | 0.093 |
| Colon:Chronic inflammation:Ulcerative colitis | 68 | 7 | 0.60 | 0.116 |
| Lung:Small cell carcinoma:Primary malignant neoplasm of lung | 113 | 8 | 0.58 | 0.108 |
| Pancreas:Adenocarcinoma:Primary malignant neoplasm of pancreas | 55 | 5 | 0.63 | 0.136 |
| Breast:Infiltrating duct and lobular carcinoma:Primary malignant neoplasm of female breast | 42 | 13 | 0.52 | 0.085 |
| bronchial brushings:Asthma | 12 | 17 | 0.50 | 0.075 |
| White blood cell:Acute myeloid leukemia, minimal differentiation, FAB M0 | 33 | 8 | 0.56 | 0.108 |
| Skin:Inflammation:Allergic dermatitis | 51 | 11 | 0.53 | 0.092 |
| Breast:Infiltrating duct carcinoma:Primary malignant neoplasm of female breast | 130 | 13 | 0.52 | 0.085 |
| Colon epithelial cell:Adenocarcinoma:Primary malignant neoplasm of colon | 24 | 2 | 0.75 | 0.202 |
| Ileum:Chronic inflammation:Crohns disease | 27 | 10 | 0.54 | 0.096 |
| Lung:Dilatation:Bronchiectasis | 111 | 2 | 0.74 | 0.203 |
| Skin:Allergic dermatitis:Inflammation | 51 | 12 | 0.51 | 0.088 |
| Colon:Acute and chronic inflammation:Ulcerative colitis | 74 | 7 | 0.57 | 0.115 |
| Blood:Peripheral vascular disease | 48 | 5 | 0.60 | 0.136 |
| Ovary:Endometrioid adenocarcinoma:Primary malignant neoplasm of ovary | 80 | 3 | 0.67 | 0.173 |
| Vein:Peripheral vascular disease:Atherosclerosis | 15 | 5 | 0.60 | 0.136 |
| Ovary:Adenocarcinoma:Primary malignant neoplasm of ovary | 70 | 3 | 0.67 | 0.174 |
| Pancreas:Intraductal papillary carcinoma | 16 | 1 | 0.83 | 0.255 |
| Colon all cells:Colon active ulcerative colitis. | 23 | 4 | 0.62 | 0.152 |
| Colon:Acute and chronic inflammation:Crohns disease | 72 | 10 | 0.50 | 0.095 |
| T lymphocyte:Rheumatoid arthritis | 10 | 17 | 0.46 | 0.073 |
| Soft tissues:Malignant melanoma:Secondary malignant neoplasm of connective and other soft tissues | 10 | 2 | 0.72 | 0.207 |
| Liver:Hepatocellular carcinoma:Primary malignant neoplasm of liver | 44 | 3 | 0.65 | 0.174 |
| Breast Epithelial cell:Primary malignant neoplasm of female breast | 11 | 13 | 0.46 | 0.082 |
| Blood:Chronic obstructive lung disease | 60 | 11 | 0.48 | 0.089 |
| Kidney:Chronic inflammation:Chronic pyelonephritis | 71 | 10 | 0.48 | 0.094 |
| Stomach:Adenocarcinoma:Primary malignant neoplasm of stomach | 18 | 5 | 0.56 | 0.135 |
| Ovary:Endometrioid adenocarcinoma:Secondary malignant neoplasm of ovary | 70 | 3 | 0.63 | 0.175 |
| Coronary artery:Atherosclerosis:Coronary artery disease | 22 | 25 | 0.40 | 0.059 |
| Ovary:Papillary serous adenocarcinoma:Primary malignant neoplasm of ovary | 92 | 3 | 0.63 | 0.175 |
| Ovary:Adenocarcinoma:Secondary malignant neoplasm of ovary | 71 | 3 | 0.62 | 0.175 |
| Left ventricle:Dilatation:Congestive cardiomyopathy | 71 | 5 | 0.54 | 0.134 |
| Colon all cells:Inflamed | 23 | 2 | 0.69 | 0.210 |
| Subcutaneous adipose tissue:Metabolic syndrome X | 36 | 8 | 0.48 | 0.104 |
| Ovary:Carcinoma:Primary malignant neoplasm of ovary | 69 | 3 | 0.62 | 0.175 |
| Ovary:Mucinous cystadenocarcinoma:Primary malignant neoplasm of ovary | 74 | 3 | 0.62 | 0.175 |
| Ovary:Serous cystadenocarcinoma:Primary malignant neoplasm of ovary | 77 | 3 | 0.62 | 0.175 |
| Ascending aorta:Atherosclerosis:Atherosclerosis of aorta | 31 | 4 | 0.57 | 0.151 |
| Rectum:Chronic inflammation | 31 | 9 | 0.46 | 0.096 |
| Blood:Allergic dermatitis | 53 | 11 | 0.44 | 0.086 |
| T lymphocyte:Crohns disease | 11 | 10 | 0.42 | 0.088 |
| Prostate:Adenocarcinoma:Primary malignant neoplasm of prostate | 112 | 6 | 0.47 | 0.118 |
| White blood cell:Systemic lupus erythematosus | 93 | 5 | 0.49 | 0.131 |
| Colon:Adenocarcinoma:Primary malignant neoplasm of colon | 76 | 2 | 0.65 | 0.213 |
| Soft tissues:Atypical lipoma:Neoplasm of uncertain behavior of connective and other soft tissues | 6 | 1 | 0.77 | 0.277 |
| Blood:Depressive disorder | 127 | 7 | 0.43 | 0.106 |
| Soft tissues:Osteosarcoma:Secondary malignant neoplasm of connective and other soft tissues | 7 | 2 | 0.63 | 0.214 |
| White blood cell:CLL - Chronic lymphocytic leukemia | 50 | 6 | 0.42 | 0.113 |
| White blood cell:Diabetes mellitus | 38 | 7 | 0.39 | 0.102 |
| Right ventricle:Myocarditis | 22 | 2 | 0.61 | 0.214 |
| Right atrium:Coronary artery disease:Recent infarct | 27 | 1 | 0.75 | 0.285 |
| Right atrium:Coronary artery disease:Old infarct | 106 | 3 | 0.51 | 0.170 |
| Sigmoid colon:Diverticulitis:Diverticulitis of colon | 36 | 1 | 0.74 | 0.287 |
| Artery:Atherosclerosis:Peripheral vascular disease | 164 | 5 | 0.41 | 0.121 |
| Spleen:Follicular lymphoma:Malignant lymphoma, non-Hodgkins type | 35 | 5 | 0.39 | 0.119 |
| Adipose tissue:Diabetes mellitus | 28 | 7 | 0.34 | 0.094 |
| White blood cell:Plasma cell leukemia | 32 | 4 | 0.40 | 0.134 |
| Sigmoid colon:Adenocarcinoma:Primary malignant neoplasm of colon | 55 | 2 | 0.55 | 0.211 |
| Right ventricle:Coronary artery disease:Old infarct | 120 | 3 | 0.45 | 0.162 |
| Stomach:Gastrointestinal stromal tumor:Primary malignant neoplasm of stomach | 17 | 5 | 0.34 | 0.111 |
| Lung:Carcinoid tumor:Neoplasm of uncertain behavior of lung | 111 | 2 | 0.54 | 0.210 |
| Kidney:Renal cell carcinoma:Primary malignant neoplasm of kidney | 80 | 1 | 0.70 | 0.295 |
| Liver:Chronic inflammation:Chronic hepatitis | 35 | 2 | 0.53 | 0.210 |
| Left ventricle:Fibrosis:Valvular heart disease | 27 | 3 | 0.43 | 0.160 |
| Left atrium:Coronary artery disease:Old infarct | 97 | 3 | 0.41 | 0.156 |
| Endometrium:Adenocarcinoma:Primary malignant neoplasm of endometrium | 14 | 1 | 0.68 | 0.298 |
| Ovary:Clear cell adenocarcinoma:Primary malignant neoplasm of ovary | 74 | 3 | 0.39 | 0.152 |
| Lung:Fibrosis:Pulmonary fibrosis | 110 | 1 | 0.67 | 0.299 |
| Cecum:Mucinous adenocarcinoma:Primary malignant neoplasm of colon | 25 | 2 | 0.47 | 0.202 |
| Skeletal muscle:Atrophy:Muscle atrophy | 33 | 2 | 0.47 | 0.202 |
| Soft tissues:Fibromatosis:Neoplasm of uncertain behavior of connective and other soft tissues | 15 | 1 | 0.66 | 0.300 |
| Gallbladder:Chronic inflammation:Chronic cholecystitis | 65 | 3 | 0.35 | 0.144 |
| Lung:Chronic inflammation:Pneumonitis | 113 | 4 | 0.28 | 0.107 |
| Skeletal muscle structure of lower limb:Atrophy:Muscle atrophy | 46 | 2 | 0.44 | 0.196 |
| White blood cell:Chronic sinusitis | 32 | 5 | 0.18 | 0.072 |
| Left ventricle:Hypertrophy:Primary hypertrophic cardiomyopathy | 56 | 2 | 0.39 | 0.185 |
| White blood cell:Wegeners granulomatosis | 55 | 1 | 0.60 | 0.302 |
| Left ventricle:Inflammation:Myocarditis | 22 | 2 | 0.35 | 0.174 |
| Soft tissues:Granulosa cell tumor, malignant:Secondary malignant neoplasm of connective and other soft tissues | 9 | 2 | 0.34 | 0.170 |
| Liver:Fibrosis:Cirrhosis of liver | 82 | 2 | 0.32 | 0.166 |
| Left ventricle:Granulomatous inflammation:Myocarditis | 22 | 2 | 0.32 | 0.165 |
| Myometrium:Endometriosis:Adenomyosis | 120 | 1 | 0.57 | 0.300 |
| Ascending colon:Mucinous adenocarcinoma:Primary malignant neoplasm of colon | 35 | 2 | 0.14 | 0.087 |
| Kidney:Glomerulosclerosis:Focal glomerulosclerosis | 64 | 1 | 0.54 | 0.297 |
| Endometrium:Mullerian mixed tumor:Primary malignant neoplasm of endometrium | 21 | 1 | 0.05 | 0.047 |
| Right ventricle:Coronary artery disease:Recent infarct | 34 | 1 | 0.53 | 0.296 |
| Left atrium:Coronary artery disease:Recent infarct | 27 | 1 | 0.49 | 0.289 |
| Ovary:Cyst | 70 | 1 | 0.48 | 0.287 |
| Duodenum:Adenocarcinoma:Primary malignant neoplasm of duodenum | 71 | 1 | 0.46 | 0.281 |
| Kidney:Chromophobe carcinoma:Primary malignant neoplasm of kidney | 64 | 1 | 0.44 | 0.277 |
| Gallbladder:Acute and chronic inflammation:Acute cholecystitis | 18 | 1 | 0.15 | 0.133 |
| Breast:Fibrocystic change, proliferative type:Fibrocystic disease of breast | 40 | 1 | 0.42 | 0.272 |
| Kidney:Clear cell adenocarcinoma:Primary malignant neoplasm of kidney | 105 | 1 | 0.42 | 0.272 |
| Uterus:Endometriosis:Adenomyosis | 50 | 1 | 0.41 | 0.268 |
| Body of stomach:Chronic inflammation:Chronic gastritis | 22 | 1 | 0.40 | 0.265 |
| Ovary:Endometriosis:Endometriosis of ovary | 75 | 1 | 0.40 | 0.263 |
| Hepatocyte:Cirrhosis of liver:Hepatitis C | 9 | 1 | 0.19 | 0.156 |
| Endometrium:Endometrioid adenocarcinoma:Primary malignant neoplasm of endometrium | 39 | 1 | 0.35 | 0.246 |
| Kidney:Wilms tumor:Primary malignant neoplasm of kidney | 69 | 1 | 0.23 | 0.186 |
| Pancreas:Chronic inflammation:Chronic pancreatitis | 20 | 1 | 0.32 | 0.231 |
| Pylorus:Chronic inflammation:Chronic gastritis | 17 | 1 | 0.28 | 0.215 |

Table S3: Drug—Indication relationships (benchmark standard)

| beclometasone | acute and chronic inflammation |
| --- | --- |
| betamethasone | acute and chronic inflammation |
| budesonide | acute and chronic inflammation |
| dexamethasone | acute and chronic inflammation |
| diflorasone | acute and chronic inflammation |
| fludroxycortide | acute and chronic inflammation |
| flunisolide | acute and chronic inflammation |
| fluticasone | acute and chronic inflammation |
| hydrocortisone | acute and chronic inflammation |
| hyoscyamine | acute and chronic inflammation |
| mometasone | acute and chronic inflammation |
| ofloxacin | Acute and chronic inflammation |
| prednisolone | acute and chronic inflammation |
| prednisone | Acute and Chronic Inflammation |
| mesalazine | acute and chronic inflammation |
| metronidazole | acute and chronic inflammation |
| sulfasalazine | acute and chronic inflammation |
| tacrolimus | acute and chronic inflammation |
| adipiodone | acute cholecystitis |
| omeprazole | Acute cholecystitis |
| azacitidine | acute myeloid leukemia, minimal differentiation, fab m0 |
| doxorubicin | acute myeloid leukemia, minimal differentiation, fab m0 |
| hydrocortisone | Acute myeloid leukemia, minimal differentiation, FAB M0 |
| imatinib | Acute myeloid leukemia, minimal differentiation, FAB M0 |
| methotrexate | Acute myeloid leukemia, minimal differentiation, FAB M0 |
| mitoxantrone | acute myeloid leukemia, minimal differentiation, fab m0 |
| tretinoin | acute myeloid leukemia, minimal differentiation, fab m0 |
| methotrexate | ACUTE PROMYELOCYTIC LEUKAEMIA |
| mitoxantrone | ACUTE PROMYELOCYTIC LEUKAEMIA |
| tretinoin | ACUTE PROMYELOCYTIC LEUKAEMIA |
| altretamine | adenocarcinoma |
| diethylstilbestrol | adenocarcinoma |
| doxorubicin | adenocarcinoma |
| estradiol | adenocarcinoma |
| etanidazole | adenocarcinoma |
| flutamide | adenocarcinoma |
| ifosfamide | adenocarcinoma |
| irinotecan | adenocarcinoma |
| levamisole | adenocarcinoma |
| nilutamide | adenocarcinoma |
| paclitaxel | adenocarcinoma |
| semustine | adenocarcinoma |
| streptozocin | adenocarcinoma |
| bromocriptine | adenoma |
| danazol | adenomyosis |
| amitriptyline | adjustment disorder |
| imipramine | adjustment disorder |
| mianserin | adjustment disorder |
| nortriptyline | adjustment disorder |
| naltrexone | alcoholic liver disease |
| astemizole | allergic dermatitis |
| cetirizine | allergic dermatitis |
| ciclosporin | allergic dermatitis |
| clemastine | allergic dermatitis |
| clioquinol | allergic dermatitis |
| clobetasol | allergic dermatitis |
| cromoglicic acid | allergic dermatitis |
| fludroxycortide | allergic dermatitis |
| fluocinonide | allergic dermatitis |
| fluticasone | allergic dermatitis |
| hydrocortisone | allergic dermatitis |
| ketotifen | allergic dermatitis |
| mepyramine | allergic dermatitis |
| mometasone | allergic dermatitis |
| prednicarbate | allergic dermatitis |
| prednisone | Allergic dermatitis |
| spironolactone | Allergic dermatitis |
| tacrolimus | allergic dermatitis |
| terfenadine | allergic dermatitis |
| tretinoin | Allergic dermatitis |
| triamcinolone | Allergic dermatitis |
| clioquinol | alzheimers disease |
| dihydroergocristine | alzheimers disease |
| galantamine | alzheimers disease |
| memantine | alzheimers disease |
| nicergoline | alzheimers disease |
| physostigmine | alzheimers disease |
| risperidone | Alzheimers disease |
| sulpiride | alzheimers disease |
| tacrine | alzheimers disease |
| velnacrine | alzheimers disease |
| alprostadil | arteriolosclerosis |
| buflomedil | arteriolosclerosis |
| isradipine | arteriolosclerosis |
| pentoxifylline | arteriolosclerosis |
| theobromine | arteriolosclerosis |
| ambroxol | asthma |
| aminophylline | asthma |
| atropine | asthma |
| bambuterol | asthma |
| beclometasone | asthma |
| budesonide | asthma |
| cetirizine | asthma |
| clemastine | asthma |
| cromoglicic acid | asthma |
| dexamethasone | asthma |
| diprophylline | asthma |
| fenoterol | asthma |
| flunisolide | asthma |
| fluticasone | asthma |
| guaifenesin | asthma |
| hydrocortisone | asthma |
| isoetarine | asthma |
| ketotifen | asthma |
| khellin | asthma |
| methacholine chloride | asthma |
| methylprednisolone | asthma |
| metronidazole | Asthma |
| mometasone | asthma |
| orciprenaline | asthma |
| prednisolone | asthma |
| prednisone | Asthma |
| proxyphylline | asthma |
| salbutamol | asthma |
| terbutaline | asthma |
| theophylline | asthma |
| triamcinolone | asthma |
| troleandomycin | asthma |
| alprostadil | Atherosclerosis |
| fluvastatin | atherosclerosis |
| gemfibrozil | Atherosclerosis |
| isosorbide | atherosclerosis |
| lovastatin | Atherosclerosis |
| nicotinic acid | atherosclerosis |
| simvastatin | Atherosclerosis |
| alprostadil | atherosclerosis |
| bezafibrate | atherosclerosis |
| buflomedil | atherosclerosis |
| isradipine | atherosclerosis |
| pentoxifylline | atherosclerosis |
| simvastatin | atherosclerosis |
| theobromine | atherosclerosis |
| alprostadil | atherosclerosis of aorta |
| buflomedil | atherosclerosis of aorta |
| fluvastatin | atherosclerosis of aorta |
| isosorbide | atherosclerosis of aorta |
| isradipine | atherosclerosis of aorta |
| nicotinic acid | atherosclerosis of aorta |
| pentoxifylline | atherosclerosis of aorta |
| theobromine | atherosclerosis of aorta |
| estradiol | atrophy |
| hydrocortisone | Benign neoplasm of liver |
| methylprednisolone | Benign neoplasm of liver |
| prednisolone | Benign neoplasm of liver |
| prednisone | Benign neoplasm of liver |
| tacrolimus | Benign neoplasm of liver |
| alfuzosin | benign prostatic hypertrophy |
| doxazosin | benign prostatic hypertrophy |
| finasteride | benign prostatic hypertrophy |
| naftopidil | Benign prostatic hypertrophy |
| terazosin | benign prostatic hypertrophy |
| amitriptyline | bipolar disorder |
| bupropion | bipolar disorder |
| desipramine | bipolar disorder |
| fluoxetine | bipolar disorder |
| imipramine | bipolar disorder |
| molindone | bipolar disorder |
| phenelzine | bipolar disorder |
| risperidone | bipolar disorder |
| sulpiride | bipolar disorder |
| topiramate | bipolar disorder |
| triflupromazine | bipolar disorder |
| trimipramine | bipolar disorder |
| valproic acid | bipolar disorder |
| aztreonam | bronchiectasis |
| prednisolone | Bronchiectasis |
| prednisone | Bronchiectasis |
| theophylline | Bronchiectasis |
| altretamine | carcinoma |
| aminoglutethimide | carcinoma |
| cyproterone | carcinoma |
| doxorubicin | carcinoma |
| flutamide | carcinoma |
| irinotecan | carcinoma |
| paclitaxel | carcinoma |
| streptozocin | carcinoma |
| acetylsalicylic acid | Cerebral degeneration |
| memantine | Cerebral degeneration |
| risperidone | Cerebral degeneration |
| simvastatin | Cerebral degeneration |
| sirolimus | chromophobe carcinoma |
| gabapentin | Chronic cholecystitis |
| hyoscyamine | Chronic cholecystitis |
| omeprazole | Chronic cholecystitis |
| paracetamol | Chronic cholecystitis |
| scopolamine | Chronic cholecystitis |
| famotidine | chronic gastritis |
| omeprazole | chronic gastritis |
| proglumide | chronic gastritis |
| prednisolone | Chronic hepatitis |
| prednisone | Chronic hepatitis |
| ribavirin | chronic hepatitis |
| ofloxacin | Chronic inflammation |
| budesonide | chronic inflammation |
| ciclosporin | chronic inflammation |
| clobetasol | chronic inflammation |
| famotidine | chronic inflammation |
| fluocinonide | chronic inflammation |
| meropenem | chronic inflammation |
| mesalazine | chronic inflammation |
| methotrexate | chronic inflammation |
| methoxsalen | chronic inflammation |
| metronidazole | chronic inflammation |
| mometasone | chronic inflammation |
| ofloxacin | chronic inflammation |
| omeprazole | chronic inflammation |
| sulfasalazine | chronic inflammation |
| tacrolimus | chronic inflammation |
| triamcinolone | chronic inflammation |
| ambroxol | chronic obstructive lung disease |
| aminophylline | chronic obstructive lung disease |
| beclometasone | chronic obstructive lung disease |
| budesonide | chronic obstructive lung disease |
| clenbuterol | chronic obstructive lung disease |
| fenoterol | chronic obstructive lung disease |
| fluticasone | chronic obstructive lung disease |
| methylprednisolone | Chronic obstructive lung disease |
| orciprenaline | chronic obstructive lung disease |
| prednisolone | Chronic obstructive lung disease |
| prednisone | Chronic obstructive lung disease |
| salbutamol | chronic obstructive lung disease |
| terbutaline | chronic obstructive lung disease |
| theophylline | chronic obstructive lung disease |
| gabexate | chronic pancreatitis |
| amikacin | chronic pyelonephritis |
| ampicillin | chronic pyelonephritis |
| cefaclor | chronic pyelonephritis |
| cefadroxil | chronic pyelonephritis |
| cefalexin | chronic pyelonephritis |
| cefalotin | chronic pyelonephritis |
| cefamandole | chronic pyelonephritis |
| cefazolin | chronic pyelonephritis |
| cefotiam | chronic pyelonephritis |
| cefuroxime | chronic pyelonephritis |
| chlortetracycline | chronic pyelonephritis |
| cinoxacin | chronic pyelonephritis |
| ciprofloxacin | chronic pyelonephritis |
| colistin | chronic pyelonephritis |
| dicloxacillin | chronic pyelonephritis |
| flumequine | chronic pyelonephritis |
| kanamycin | chronic pyelonephritis |
| latamoxef | chronic pyelonephritis |
| lomefloxacin | chronic pyelonephritis |
| nalidixic acid | chronic pyelonephritis |
| norfloxacin | chronic pyelonephritis |
| ofloxacin | chronic pyelonephritis |
| phenazopyridine | chronic pyelonephritis |
| pivampicillin | chronic pyelonephritis |
| rolitetracycline | chronic pyelonephritis |
| ticarcillin | chronic pyelonephritis |
| trimethoprim | chronic pyelonephritis |
| amoxicillin | chronic sinusitis |
| bacampicillin | chronic sinusitis |
| cefaclor | chronic sinusitis |
| cefadroxil | chronic sinusitis |
| cefalexin | chronic sinusitis |
| cefamandole | chronic sinusitis |
| cefmetazole | chronic sinusitis |
| cefotaxime | chronic sinusitis |
| cefotiam | chronic sinusitis |
| cefuroxime | chronic sinusitis |
| demeclocycline | chronic sinusitis |
| dicloxacillin | chronic sinusitis |
| erythromycin | chronic sinusitis |
| guaifenesin | chronic sinusitis |
| josamycin | chronic sinusitis |
| lomefloxacin | chronic sinusitis |
| metacycline | chronic sinusitis |
| mometasone | chronic sinusitis |
| ofloxacin | chronic sinusitis |
| pivampicillin | chronic sinusitis |
| roxithromycin | chronic sinusitis |
| thiamphenicol | chronic sinusitis |
| tobramycin | chronic sinusitis |
| ribavirin | Cirrhosis of liver |
| spironolactone | Cirrhosis of liver |
| canrenoic acid | cirrhosis of liver |
| etacrynic acid | cirrhosis of liver |
| spironolactone | cirrhosis of liver |
| torasemide | cirrhosis of liver |
| triamterene | cirrhosis of liver |
| diethylstilbestrol | clear cell adenocarcinoma |
| hydrocortisone | CLL - Chronic lymphocytic leukemia |
| ifosfamide | CLL - Chronic lymphocytic leukemia |
| imatinib | CLL - Chronic lymphocytic leukemia |
| methotrexate | CLL - Chronic lymphocytic leukemia |
| methylprednisolone | CLL - Chronic lymphocytic leukemia |
| mitoxantrone | CLL - Chronic lymphocytic leukemia |
| prednisolone | CLL - Chronic lymphocytic leukemia |
| prednisone | CLL - Chronic lymphocytic leukemia |
| budesonide | colon active ulcerative colitis. |
| mesalazine | colon active ulcerative colitis. |
| metronidazole | Colon active ulcerative colitis. |
| prednisone | Colon active ulcerative colitis. |
| sulfasalazine | colon active ulcerative colitis. |
| tacrolimus | colon active ulcerative colitis. |
| acetylsalicylic acid | Congestive cardiomyopathy |
| isosorbide | Congestive cardiomyopathy |
| metoprolol | Congestive cardiomyopathy |
| simvastatin | Congestive cardiomyopathy |
| spironolactone | Congestive cardiomyopathy |
| alprenolol | congestive cardiomyopathy |
| amrinone | congestive cardiomyopathy |
| bisoprolol | congestive cardiomyopathy |
| digoxin | congestive cardiomyopathy |
| dobutamine | congestive cardiomyopathy |
| hydralazine | congestive cardiomyopathy |
| lanatoside c | congestive cardiomyopathy |
| metoprolol | congestive cardiomyopathy |
| milrinone | congestive cardiomyopathy |
| minoxidil | congestive cardiomyopathy |
| nitrendipine | congestive cardiomyopathy |
| xamoterol | congestive cardiomyopathy |
| acetylsalicylic acid | Coronary artery disease |
| alprostadil | coronary artery disease |
| bepridil | coronary artery disease |
| bisoprolol | coronary artery disease |
| buflomedil | coronary artery disease |
| clofibrate | coronary artery disease |
| dilazep | coronary artery disease |
| diltiazem | coronary artery disease |
| dipyridamole | coronary artery disease |
| fasudil | coronary artery disease |
| felodipine | coronary artery disease |
| fendiline | coronary artery disease |
| fluvastatin | coronary artery disease |
| gemfibrozil | coronary artery disease |
| isosorbide | Coronary artery disease |
| isradipine | coronary artery disease |
| khellin | coronary artery disease |
| lanatoside c | coronary artery disease |
| lidoflazine | coronary artery disease |
| lovastatin | coronary artery disease |
| metoprolol | coronary artery disease |
| molsidomine | coronary artery disease |
| naftidrofuryl | coronary artery disease |
| nifedipine | coronary artery disease |
| nimodipine | coronary artery disease |
| papaverine | coronary artery disease |
| pentoxifylline | coronary artery disease |
| perhexiline | coronary artery disease |
| phenoxybenzamine | coronary artery disease |
| prenylamine | coronary artery disease |
| simvastatin | coronary artery disease |
| spironolactone | Coronary artery disease |
| theobromine | coronary artery disease |
| ticlopidine | coronary artery disease |
| trimetazidine | coronary artery disease |
| xamoterol | coronary artery disease |
| ciclosporin | coronary artery disease |
| dobutamine | coronary artery disease |
| fluvastatin | coronary artery disease |
| isosorbide | coronary artery disease |
| nicotinic acid | coronary artery disease |
| triflusal | coronary artery disease |
| budesonide | crohns disease |
| hydrocortisone | Crohns disease |
| loperamide | crohns disease |
| mesalazine | crohns disease |
| methotrexate | Crohns disease |
| methylprednisolone | Crohns disease |
| metronidazole | crohns disease |
| omeprazole | Crohns disease |
| prednisolone | Crohns disease |
| prednisone | Crohns disease |
| sulfasalazine | Crohns disease |
| nitrofurantoin | Cyst |
| paclitaxel | Cyst |
| trimethoprim | Cyst |
| levodopa | degeneration |
| lisuride | degeneration |
| nicergoline | degeneration |
| pergolide | degeneration |
| piribedil | degeneration |
| selegiline | degeneration |
| terguride | degeneration |
| verteporfin | degeneration |
| amitriptyline | depressive disorder |
| amoxapine | depressive disorder |
| brompheniramine | depressive disorder |
| bupropion | depressive disorder |
| buspirone | depressive disorder |
| citalopram | depressive disorder |
| clomipramine | depressive disorder |
| desipramine | depressive disorder |
| dosulepin | depressive disorder |
| doxepin | depressive disorder |
| fluoxetine | depressive disorder |
| fluvoxamine | depressive disorder |
| imipramine | depressive disorder |
| isocarboxazid | depressive disorder |
| maprotiline | depressive disorder |
| mianserin | depressive disorder |
| minaprine | depressive disorder |
| nortriptyline | depressive disorder |
| paroxetine | depressive disorder |
| phenelzine | depressive disorder |
| protriptyline | depressive disorder |
| risperidone | depressive disorder |
| selegiline | depressive disorder |
| serotonin | depressive disorder |
| tranylcypromine | depressive disorder |
| trazodone | depressive disorder |
| trimipramine | depressive disorder |
| zimeldine | depressive disorder |
| acetohexamide | diabetes mellitus |
| bezafibrate | diabetes mellitus |
| chlorpropamide | diabetes mellitus |
| gabapentin | Diabetes mellitus |
| glibenclamide | diabetes mellitus |
| gliclazide | diabetes mellitus |
| glimepiride | diabetes mellitus |
| glipizide | diabetes mellitus |
| gliquidone | diabetes mellitus |
| metformin | diabetes mellitus |
| phenformin | diabetes mellitus |
| pioglitazone | diabetes mellitus |
| repaglinide | diabetes mellitus |
| rosiglitazone | diabetes mellitus |
| simvastatin | Diabetes mellitus |
| tolazamide | diabetes mellitus |
| tolbutamide | diabetes mellitus |
| troglitazone | diabetes mellitus |
| hyoscyamine | diverticulitis |
| loperamide | diverticulitis |
| mesalazine | diverticulitis |
| scopolamine | diverticulitis |
| metronidazole | Diverticulitis of colon |
| aminophylline | emphysema |
| salbutamol | emphysema |
| theophylline | emphysema |
| bambuterol | emphysema |
| orciprenaline | emphysema |
| terbutaline | emphysema |
| torasemide | end stage kidney disease |
| megestrol | endometrial hyperplasia |
| norethisterone | endometrial hyperplasia |
| progesterone | endometrial hyperplasia |
| tamoxifen | endometrial hyperplasia |
| clomifene | endometrioid adenocarcinoma |
| estradiol | endometrioid adenocarcinoma |
| megestrol | endometrioid adenocarcinoma |
| progesterone | endometrioid adenocarcinoma |
| raloxifene | endometrioid adenocarcinoma |
| tamoxifen | endometrioid adenocarcinoma |
| danazol | endometriosis |
| estradiol | endometriosis |
| norethisterone | endometriosis |
| danazol | endometriosis of ovary |
| pioglitazone | fatty liver |
| troglitazone | fatty liver |
| acetylsalicylic acid | fibrin thrombus |
| triflusal | fibrin thrombus |
| danazol | fibrocystic change, proliferative type |
| danazol | fibrocystic disease of breast |
| etanidazole | fibrosarcoma |
| meropenem | Fibrosis |
| tobramycin | Fibrosis |
| tyloxapol | fibrosis |
| chlorambucil | focal glomerulosclerosis |
| ciclosporin | focal glomerulosclerosis |
| carbimazole | follicular adenoma |
| etanidazole | follicular adenoma |
| liothyronine | follicular adenoma |
| chlorambucil | follicular lymphoma |
| daunorubicin | follicular lymphoma |
| prednisone | follicular lymphoma |
| vidarabine | follicular lymphoma |
| imatinib | gastrointestinal stromal tumor |
| semustine | gastrointestinal stromal tumor |
| prednisone | Granulomatous inflammation |
| levothyroxine sodium | hashimoto thyroiditis |
| liothyronine | hashimoto thyroiditis |
| thiamazole | hashimoto thyroiditis |
| ribavirin | hepatitis c |
| alfuzosin | hyperplasia |
| doxazosin | hyperplasia |
| finasteride | hyperplasia |
| naftopidil | hyperplasia |
| prednisolone | hyperplasia |
| prednisone | hyperplasia |
| terazosin | hyperplasia |
| alfuzosin | hyperplastic glandular epithelium |
| doxazosin | hyperplastic glandular epithelium |
| finasteride | hyperplastic glandular epithelium |
| terazosin | hyperplastic glandular epithelium |
| levothyroxine sodium | Hyperplastic goiter |
| phenoxybenzamine | hypertrophy |
| budesonide | inflamed |
| mesalazine | inflamed |
| methotrexate | inflammation |
| methylprednisolone | inflammation |
| nabumetone | inflammation |
| naproxen | inflammation |
| ofloxacin | Inflammation |
| omeprazole | inflammation |
| prednisolone | inflammation |
| prednisone | inflammation |
| benzydamine | inflammation |
| capsaicin | inflammation |
| cetirizine | inflammation |
| ciclosporin | inflammation |
| clobetasol | inflammation |
| fluorometholone | inflammation |
| fluticasone | inflammation |
| gemfibrozil | inflammation |
| hydrocortisone | inflammation |
| mefenamic acid | inflammation |
| nimesulide | inflammation |
| oxaprozin | inflammation |
| prednisone | inflammation |
| rimexolone | inflammation |
| tacrolimus | inflammation |
| terfenadine | inflammation |
| tamoxifen | intraductal carcinoma |
| paclitaxel | Intraductal papillary carcinoma |
| diazoxide | islet cell carcinoma |
| streptozocin | islet cell carcinoma |
| paclitaxel | large cell carcinoma |
| raloxifene | leiomyoma |
| ifosfamide | Liposarcoma |
| methotrexate | Liposarcoma |
| hydrocortisone | Malignant lymphoma of extranodal AND/OR solid organ site |
| ifosfamide | Malignant lymphoma of extranodal AND/OR solid organ site |
| methotrexate | Malignant lymphoma of extranodal AND/OR solid organ site |
| prednisolone | Malignant lymphoma of extranodal AND/OR solid organ site |
| prednisone | Malignant lymphoma of extranodal AND/OR solid organ site |
| ifosfamide | Malignant lymphoma, non-Hodgkins type |
| methotrexate | Malignant lymphoma, non-Hodgkins type |
| methylprednisolone | Malignant lymphoma, non-Hodgkins type |
| mitoxantrone | Malignant lymphoma, non-Hodgkins type |
| prednisolone | Malignant lymphoma, non-Hodgkins type |
| prednisone | Malignant lymphoma, non-Hodgkins type |
| vinblastine | Malignant lymphoma, non-Hodgkins type |
| dacarbazine | malignant melanoma |
| levamisole | malignant melanoma |
| lomustine | malignant melanoma |
| paclitaxel | malignant melanoma |
| semustine | malignant melanoma |
| benfluorex | metabolic syndrome x |
| chlorpropamide | metabolic syndrome x |
| clofibrate | metabolic syndrome x |
| diazoxide | metabolic syndrome x |
| fenofibrate | metabolic syndrome x |
| glibenclamide | metabolic syndrome x |
| glimepiride | metabolic syndrome x |
| metformin | metabolic syndrome x |
| orlistat | metabolic syndrome x |
| pioglitazone | metabolic syndrome x |
| rosiglitazone | metabolic syndrome x |
| tolbutamide | metabolic syndrome x |
| troglitazone | metabolic syndrome x |
| clenbuterol | muscle atrophy |
| prednisolone | Muscle atrophy |
| chlorambucil | mycosis fungoides |
| daunorubicin | mycosis fungoides |
| methoxsalen | mycosis fungoides |
| prednisone | mycosis fungoides |
| vinblastine | mycosis fungoides |
| acetylsalicylic acid | Myocarditis |
| isosorbide | Myocarditis |
| metoprolol | Myocarditis |
| simvastatin | Myocarditis |
| spironolactone | Myocarditis |
| lanatoside c | myocarditis |
| paclitaxel | Neoplasm of uncertain behavior of connective and other soft tissues |
| methylprednisolone | Neoplasm of uncertain behavior of lung |
| prednisolone | Neoplasm of uncertain behavior of lung |
| prednisone | Neoplasm of uncertain behavior of lung |
| tacrolimus | Neoplasm of uncertain behavior of lung |
| galantamine | neurofibrillary degeneration |
| memantine | neurofibrillary degeneration |
| nicergoline | neurofibrillary degeneration |
| tacrine | neurofibrillary degeneration |
| alfuzosin | nodular hyperplasia |
| doxazosin | nodular hyperplasia |
| finasteride | nodular hyperplasia |
| terazosin | nodular hyperplasia |
| danazol | nonproliferative fibrocystic change |
| ciclosporin | old infarct |
| dobutamine | old infarct |
| isosorbide | old infarct |
| nicotinic acid | old infarct |
| triflusal | old infarct |
| aceclofenac | osteoarthritis |
| celecoxib | osteoarthritis |
| dexibuprofen | osteoarthritis |
| diclofenac | osteoarthritis |
| diflunisal | osteoarthritis |
| etodolac | osteoarthritis |
| fenoprofen | osteoarthritis |
| flurbiprofen | osteoarthritis |
| gabapentin | Osteoarthritis |
| indoprofen | osteoarthritis |
| ketoprofen | osteoarthritis |
| leflunomide | Osteoarthritis |
| meclofenamic acid | osteoarthritis |
| mefenamic acid | osteoarthritis |
| methotrexate | Osteoarthritis |
| methylprednisolone | Osteoarthritis |
| nabumetone | osteoarthritis |
| naproxen | osteoarthritis |
| nimesulide | osteoarthritis |
| oxaprozin | osteoarthritis |
| paracetamol | osteoarthritis |
| piroxicam | osteoarthritis |
| prednisolone | Osteoarthritis |
| prednisone | Osteoarthritis |
| rofecoxib | osteoarthritis |
| sulfasalazine | Osteoarthritis |
| sulindac | osteoarthritis |
| tenoxicam | osteoarthritis |
| tiaprofenic acid | osteoarthritis |
| tolmetin | osteoarthritis |
| triamcinolone | osteoarthritis |
| valdecoxib | osteoarthritis |
| methotrexate | osteosarcoma |
| orlistat | overweight |
| amantadine | parkinsons disease |
| apomorphine | parkinsons disease |
| benserazide | parkinsons disease |
| biperiden | parkinsons disease |
| bromocriptine | parkinsons disease |
| dantrolene | parkinsons disease |
| dopamine | parkinsons disease |
| harmine | parkinsons disease |
| levodopa | parkinsons disease |
| lisuride | parkinsons disease |
| memantine | parkinsons disease |
| metixene | parkinsons disease |
| orphenadrine | parkinsons disease |
| pergolide | parkinsons disease |
| piribedil | parkinsons disease |
| prochlorperazine | parkinsons disease |
| procyclidine | parkinsons disease |
| profenamine | parkinsons disease |
| quinpirole | parkinsons disease |
| salsolinol | parkinsons disease |
| selegiline | parkinsons disease |
| spiperone | parkinsons disease |
| terguride | parkinsons disease |
| tiapride | parkinsons disease |
| trihexyphenidyl | parkinsons disease |
| alprostadil | peripheral vascular disease |
| buflomedil | peripheral vascular disease |
| flunarizine | peripheral vascular disease |
| iloprost | peripheral vascular disease |
| paclitaxel | peripheral vascular disease |
| isosorbide | peripheral vascular disease |
| nicotinic acid | peripheral vascular disease |
| dexamethasone | plasma cell leukemia |
| doxorubicin | plasma cell leukemia |
| methotrexate | Plasma cell leukemia |
| thalidomide | plasma cell leukemia |
| amoxicillin | pneumonitis |
| bacampicillin | pneumonitis |
| cefaclor | pneumonitis |
| cefadroxil | pneumonitis |
| cefalexin | pneumonitis |
| cefamandole | pneumonitis |
| cefixime | pneumonitis |
| cefmetazole | pneumonitis |
| cefotaxime | pneumonitis |
| cefotiam | pneumonitis |
| cefuroxime | pneumonitis |
| demeclocycline | pneumonitis |
| dicloxacillin | pneumonitis |
| erythromycin | pneumonitis |
| guaifenesin | pneumonitis |
| josamycin | pneumonitis |
| lomefloxacin | pneumonitis |
| meropenem | pneumonitis |
| metacycline | pneumonitis |
| ofloxacin | pneumonitis |
| pivampicillin | pneumonitis |
| prednisone | pneumonitis |
| roxithromycin | pneumonitis |
| thiamphenicol | pneumonitis |
| tobramycin | pneumonitis |
| acetylsalicylic acid | Primary hypertrophic cardiomyopathy |
| isosorbide | Primary hypertrophic cardiomyopathy |
| metoprolol | Primary hypertrophic cardiomyopathy |
| simvastatin | Primary hypertrophic cardiomyopathy |
| spironolactone | Primary hypertrophic cardiomyopathy |
| altretamine | primary malignant neoplasm |
| doxorubicin | primary malignant neoplasm |
| irinotecan | primary malignant neoplasm |
| paclitaxel | primary malignant neoplasm |
| methotrexate | Primary malignant neoplasm of bladder |
| paclitaxel | Primary malignant neoplasm of bladder |
| irinotecan | primary malignant neoplasm of colon |
| levamisole | primary malignant neoplasm of colon |
| omeprazole | Primary malignant neoplasm of duodenum |
| paclitaxel | Primary malignant neoplasm of endometrium |
| aminoglutethimide | primary malignant neoplasm of female breast |
| doxorubicin | primary malignant neoplasm of female breast |
| exemestane | primary malignant neoplasm of female breast |
| fulvestrant | primary malignant neoplasm of female breast |
| irinotecan | primary malignant neoplasm of female breast |
| letrozole | primary malignant neoplasm of female breast |
| megestrol | primary malignant neoplasm of female breast |
| methotrexate | Primary malignant neoplasm of female breast |
| methylprednisolone | Primary malignant neoplasm of female breast |
| mitoxantrone | primary malignant neoplasm of female breast |
| paclitaxel | primary malignant neoplasm of female breast |
| raloxifene | primary malignant neoplasm of female breast |
| tamoxifen | primary malignant neoplasm of female breast |
| methylprednisolone | Primary malignant neoplasm of kidney |
| hydrocortisone | Primary malignant neoplasm of liver |
| methylprednisolone | Primary malignant neoplasm of liver |
| prednisolone | Primary malignant neoplasm of liver |
| prednisone | Primary malignant neoplasm of liver |
| tacrolimus | Primary malignant neoplasm of liver |
| doxorubicin | primary malignant neoplasm of lung |
| ifosfamide | primary malignant neoplasm of lung |
| irinotecan | primary malignant neoplasm of lung |
| methylprednisolone | Primary malignant neoplasm of lung |
| paclitaxel | primary malignant neoplasm of lung |
| prednisolone | Primary malignant neoplasm of lung |
| prednisone | Primary malignant neoplasm of lung |
| tacrolimus | Primary malignant neoplasm of lung |
| altretamine | primary malignant neoplasm of ovary |
| doxorubicin | primary malignant neoplasm of ovary |
| irinotecan | primary malignant neoplasm of ovary |
| paclitaxel | primary malignant neoplasm of ovary |
| diazoxide | primary malignant neoplasm of pancreas |
| ifosfamide | primary malignant neoplasm of pancreas |
| irinotecan | primary malignant neoplasm of pancreas |
| paclitaxel | primary malignant neoplasm of pancreas |
| semustine | primary malignant neoplasm of pancreas |
| streptozocin | primary malignant neoplasm of pancreas |
| estradiol | primary malignant neoplasm of prostate |
| flutamide | primary malignant neoplasm of prostate |
| hydrocortisone | Primary malignant neoplasm of prostate |
| mitoxantrone | Primary malignant neoplasm of prostate |
| nilutamide | primary malignant neoplasm of prostate |
| thalidomide | Primary malignant neoplasm of prostate |
| irinotecan | Primary malignant neoplasm of rectum |
| doxorubicin | primary malignant neoplasm of stomach |
| irinotecan | primary malignant neoplasm of stomach |
| omeprazole | Primary malignant neoplasm of stomach |
| paclitaxel | primary malignant neoplasm of stomach |
| methotrexate | Primary malignant neoplasm of uterine cervix |
| paclitaxel | Primary malignant neoplasm of uterine cervix |
| alclometasone | psoriasis |
| allantoin | psoriasis |
| beclometasone | psoriasis |
| betamethasone | psoriasis |
| budesonide | psoriasis |
| ciclosporin | psoriasis |
| clioquinol | psoriasis |
| clobetasol | psoriasis |
| colecalciferol | psoriasis |
| dexamethasone | psoriasis |
| diflorasone | psoriasis |
| fludroxycortide | psoriasis |
| fluocinonide | psoriasis |
| fluorometholone | psoriasis |
| fluticasone | psoriasis |
| halcinonide | psoriasis |
| hydrocortisone | psoriasis |
| methotrexate | psoriasis |
| methoxsalen | psoriasis |
| methylprednisolone | psoriasis |
| mometasone | psoriasis |
| prednisolone | psoriasis |
| prednisone | psoriasis |
| tretinoin | psoriasis |
| triamcinolone | psoriasis |
| orciprenaline | pulmonary emphysema |
| prednisolone | Pulmonary fibrosis |
| simvastatin | Recent infarct |
| sirolimus | renal cell carcinoma |
| aceclofenac | rheumatoid arthritis |
| acemetacin | rheumatoid arthritis |
| aminophenazone | rheumatoid arthritis |
| azathioprine | rheumatoid arthritis |
| betamethasone | rheumatoid arthritis |
| celecoxib | rheumatoid arthritis |
| chloroquine | rheumatoid arthritis |
| ciclosporin | rheumatoid arthritis |
| dexamethasone | rheumatoid arthritis |
| dexibuprofen | rheumatoid arthritis |
| diclofenac | rheumatoid arthritis |
| diflunisal | rheumatoid arthritis |
| etodolac | rheumatoid arthritis |
| fenoprofen | rheumatoid arthritis |
| flurbiprofen | rheumatoid arthritis |
| gabapentin | Rheumatoid arthritis |
| hydrocortisone | rheumatoid arthritis |
| indometacin | rheumatoid arthritis |
| indoprofen | rheumatoid arthritis |
| ketoprofen | rheumatoid arthritis |
| leflunomide | rheumatoid arthritis |
| meclofenamic acid | rheumatoid arthritis |
| mefenamic acid | rheumatoid arthritis |
| methotrexate | rheumatoid arthritis |
| methylprednisolone | rheumatoid arthritis |
| nabumetone | rheumatoid arthritis |
| naproxen | rheumatoid arthritis |
| niflumic acid | rheumatoid arthritis |
| nimesulide | Rheumatoid arthritis |
| omeprazole | Rheumatoid arthritis |
| oxaprozin | rheumatoid arthritis |
| oxyphenbutazone | rheumatoid arthritis |
| paracetamol | Rheumatoid arthritis |
| piroxicam | rheumatoid arthritis |
| prednisolone | rheumatoid arthritis |
| prednisone | rheumatoid arthritis |
| rimexolone | rheumatoid arthritis |
| rofecoxib | Rheumatoid arthritis |
| sulfasalazine | rheumatoid arthritis |
| sulindac | rheumatoid arthritis |
| tacrolimus | rheumatoid arthritis |
| tenoxicam | rheumatoid arthritis |
| tiaprofenic acid | rheumatoid arthritis |
| tolmetin | rheumatoid arthritis |
| triamcinolone | rheumatoid arthritis |
| dacarbazine | sarcoma |
| paclitaxel | sarcoma |
| vinblastine | sarcoma |
| biperiden | schizophrenia |
| bromperidol | schizophrenia |
| chlorpromazine | schizophrenia |
| chlorprothixene | schizophrenia |
| clozapine | schizophrenia |
| dopamine | schizophrenia |
| fluspirilene | schizophrenia |
| mesoridazine | schizophrenia |
| molindone | schizophrenia |
| perphenazine | schizophrenia |
| pimozide | schizophrenia |
| piperacetazine | schizophrenia |
| prochlorperazine | schizophrenia |
| procyclidine | schizophrenia |
| remoxipride | schizophrenia |
| risperidone | schizophrenia |
| spiperone | schizophrenia |
| sulpiride | schizophrenia |
| thioproperazine | schizophrenia |
| thioridazine | schizophrenia |
| triflupromazine | schizophrenia |
| zuclopenthixol | schizophrenia |
| doxorubicin | secondary malignant neoplasm of connective and other soft tissues |
| paclitaxel | secondary malignant neoplasm of connective and other soft tissues |
| altretamine | secondary malignant neoplasm of ovary |
| doxorubicin | secondary malignant neoplasm of ovary |
| irinotecan | secondary malignant neoplasm of ovary |
| paclitaxel | secondary malignant neoplasm of ovary |
| orlistat | simple obesity |
| phenylpropanolamine | simple obesity |
| ifosfamide | small cell carcinoma |
| irinotecan | small cell carcinoma |
| etanidazole | squamous cell carcinoma |
| paclitaxel | squamous cell carcinoma |
| betamethasone | systemic lupus erythematosus |
| dexamethasone | systemic lupus erythematosus |
| hydralazine | systemic lupus erythematosus |
| hydrocortisone | systemic lupus erythematosus |
| methylprednisolone | systemic lupus erythematosus |
| prednisolone | systemic lupus erythematosus |
| prednisone | systemic lupus erythematosus |
| tacrolimus | Systemic lupus erythematosus |
| chlorambucil | t-cell acute lymphoblastic leukemia |
| daunorubicin | t-cell acute lymphoblastic leukemia |
| dexamethasone | t-cell acute lymphoblastic leukemia |
| doxorubicin | t-cell acute lymphoblastic leukemia |
| imatinib | t-cell acute lymphoblastic leukemia |
| methotrexate | t-cell acute lymphoblastic leukemia |
| pha-00665752 | t-cell acute lymphoblastic leukemia |
| prednisolone | T-cell acute lymphoblastic leukemia |
| prednisone | t-cell acute lymphoblastic leukemia |
| tretinoin | teratoma, benign |
| clomifene | thecoma |
| budesonide | ulcerative colitis |
| hydrocortisone | ulcerative colitis |
| loperamide | ulcerative colitis |
| mesalazine | ulcerative colitis |
| methotrexate | Ulcerative colitis |
| prednisolone | ulcerative colitis |
| prednisone | Ulcerative colitis |
| sulfasalazine | ulcerative colitis |
| tacrolimus | ulcerative colitis |
| alprostadil | Valvular heart disease |
| metoprolol | Valvular heart disease |
| prednisolone | Valvular heart disease |
| prednisone | Valvular heart disease |
| tacrolimus | Valvular heart disease |
| lanatoside c | valvular heart disease |
| prednisolone | wegeners granulomatosis |

Table S4: AUC performance and random permutation p value for three metrics using all (unfiltered) compound profiles

| **Method** | **AvgAUC** | **AvgAUC0.1** | **OneAUC** | **OneAUC0.1** |
| --- | --- | --- | --- | --- |
| KS | 0.50 (p=0.50) | 0.003 (p=0.93) | 0.48 (p=0.69) | 0.004 (p=0.97) |
| XSum | 0.50 (p=0.43) | 0.008 (p=0.11) | 0.51 (p=0.11) | 0.007 (p=0.13) |
| XCos | 0.51 (p=0.43) | 0.006 (p=0.26) | 0.51 (p=0.10) | 0.007 (p=0.16) |

Table S5: Top 20 disease-drug pairs by XSum

| **Disease signature** | **Compound** | **Xsum score** |
| --- | --- | --- |
| White blood cell:Plasma cell leukemia | pergolide | 94.12335 |
| Lung:Small cell carcinoma:Primary malignant neoplasm of lung | spiperone | 80.0817 |
| Right ventricle:Valvular heart disease:Fibrosis | ambroxol | 76.566666 |
| Kidney:Wilms tumor:Primary malignant neoplasm of kidney | spiperone | 74.34498 |
| T lymphocyte:Crohns disease | pergolide | 71.8083 |
| T lymphocyte:Crohns disease | quinpirole | 71.42198 |
| Soft tissues:Fibrosarcoma:Primary malignant neoplasm of soft tissues | spiperone | 70.35166 |
| T lymphocyte:Crohns disease | dihydroergocristine | 69.56599 |
| White blood cell:Acute myeloid leukemia, minimal differentiation, FAB M0 | pergolide | 68.023346 |
| Kidney:Wilms tumor:Primary malignant neoplasm of kidney | irinotecan | 68.020004 |
| Lung:Small cell carcinoma:Primary malignant neoplasm of lung | methotrexate | 67.66578 |
| Kidney:Wilms tumor:Primary malignant neoplasm of kidney | methotrexate | 66.58444 |
| Blood:Depressive disorder | daunorubicin | 65.575 |
| Spleen:Aberrant tissue:Extramedullary hematopoiesis of spleen | spiperone | 65.55335 |
| Endometrium:Mullerian mixed tumor:Primary malignant neoplasm of endometrium | spiperone | 65.12335 |
| Kidney:Wilms tumor:Primary malignant neoplasm of kidney | phenoxybenzamine | 64.872215 |
| White blood cell:Plasma cell leukemia | bromocriptine | 64.62499 |
| Omentum:Mullerian mixed tumor:Secondary malignant neoplasm of the omentum | methotrexate | 63.488323 |
| T lymphocyte:Crohns disease | alprostadil | 62.658672 |
| Endometrium:Mullerian mixed tumor:Primary malignant neoplasm of endometrium | methotrexate | 62.5989 |
